# *Wolbachia* prevalence in *Aedes aegypti* across five comunas of Medellín, Colombia: Implications for post-release surveillance

**DOI:** 10.1371/journal.pntd.0014630

**Published:** 2026-08-10

**Authors:** Luisa M. Barrientos, Diego F. Rincon, Frank W. Avila, Catalina Alfonso-Parra

**Affiliations:** 1 Instituto Colombiano de Medicina Tropical, Universidad CES, Sabaneta, Colombia; 2 Max Planck Tandem Group in Mosquito Reproductive Biology, Universidad de Antioquia, Medellín, Colombia; 3 Corporación Colombiana de Investigación Agropecuaria (AGROSAVIA), Mosquera, Colombia; 4 Department of Entomology, Washington State University, Pullman, Washington, United States of America; Instituto Leonidas e Maria Deane / Fundacao Oswaldo Cruz, BRAZIL

## Abstract

A promising sustainable strategy for reducing arbovirus transmission by the mosquito vector *Aedes aegypti* is the deployment of individuals transinfected with *Wolbachia pipientis*, a bacterium that inhibits replication of viruses such as dengue and is being used in population replacement programs aimed at blocking *Aedes*-borne virus transmission. Between 2017 and 2022, the World Mosquito Program released *Ae. aegypti* infected with the *w*Mel *Wolbachia* strain throughout Medellín, Colombia. Long-term *Wolbachia* surveillance following population replacement requires approaches that balance accuracy and cost while providing reliable information regarding infection persistence in targeted populations. Therefore, we assessed current *Wolbachia* prevalence in *Ae. aegypti*, surveying five comunas (geographic administrative units) of Medellín spanning ~32.66 km, using ovitrap collections and field sampling of adult mosquitoes and assessed *Wolbachia* infection using conventional PCR and quantitative PCR (qPCR) in pooled and individual samples. We found that *Wolbachia* infection remains widespread across the surveyed areas, with estimated infection frequencies ranging from ~60% to near fixation depending on sampling strategy and molecular detection method. Screening pooled adults emerging from ovitraps using conventional PCR provided a cost-effective method for confirming *Wolbachia* persistence across sites, whereas qPCR of individual field-captured adults provided more sensitive estimates of infection prevalence. Our results suggest that *Wolbachia* is broadly established in *Ae. aegypti* populations in the surveyed areas and demonstrate practical approaches for the long-term surveillance of *Wolbachia*-based vector control programs.

## Author summary

Mosquito-borne viruses such as dengue, Zika, and chikungunya are major public health problems in tropical cities worldwide. One promising strategy to reduce transmission of these viruses involves releasing *Aedes aegypti* mosquitoes infected with the bacterium *Wolbachia*. This bacterium reduces the ability of mosquitoes to transmit viruses and can spread naturally through mosquito populations. Between 2017 and 2022, the World Mosquito Program released *Wolbachia*-infected mosquitoes throughout Medellín, Colombia. In this study, we reassessed the presence of *Wolbachia* in *Aedes aegypti* across five large geographical areas of Medellín and evaluated practical methods for monitoring infection levels after mosquito releases. We collected mosquitoes using ovitraps and indoor sampling and used two molecular methods to detect *Wolbachia*. We found that *Wolbachia* infection remains widespread across the surveyed areas, with infection frequencies generally ranging from about 60% to nearly 100%. Our results also showed that screening pooled mosquito samples from ovitraps using conventional PCR provides a cost-effective way to monitor *Wolbachia* in field populations. These findings support the continued establishment of *Wolbachia* in the surveyed areas and provide guidance for long-term surveillance of mosquito control programs.

## Introduction

*Aedes aegypti* mosquitoes are the primary vectors of numerous viruses that impact human health, including dengue [1], Zika [2] and chikungunya [3] viruses. This vector species has a broad geographical distribution, being present throughout the tropics and subtropics [4], where it thrives in human-dense environments [5]. Female *Ae. aegypti* mosquitoes have a preference for human blood meals [6–8] and primarily oviposit in artificial, man-made containers [9–11], behaviors that facilitate disease transmission [12]. Although the current distribution of this vector species is vast, it is predicted to expand with increased urban development [12–14] and rising global temperatures [15,16].

In Colombia, *Ae. aegypti* is a continuing public health concern, as nearly half of the country’s population (~25 million people) reside in areas with a high risk of transmission of *Aedes* borne diseases [17,18]. Dengue epidemics in Colombia occur approximately every 3–4 years [19]. In 2024, the number of dengue cases increased threefold compared to the previous year, while the number of reported deaths doubled [20]. In Medellín, Colombia’s second largest city with a metropolitan population of more than 4 million people, *Ae. aegypti* is abundant and drives high rates of viral transmission [21]. To reduce the burden of viruses transmitted by *Ae. aegypti* in Medellín, the World Mosquito Program (WMP) performed population replacement of native *Ae. aegypti* with individuals infected with the bacterial symbiont *Wolbachia pipienti*s [22], a promising strategy to reduce arbovirus transmission.

*Wolbachia* is a maternally inherited, obligate intracellular bacterium that naturally infects 40–52% of insect species [23,24], but is not normally found *Ae. aegypti*. The *w*Mel strain of *Wolbachia,* originally isolated from *Drosophila melanogaster*, has been successfully introduced into *Ae. aegypti* [25]. *w*Mel *Wolbachia* induces cytoplasmic incompatibility, a phenomenon where uninfected females that mate with infected males do not produce viable progeny, while infected females produce viable, *Wolbachia*-infected progeny regardless of the infection status of their mates. This reproductive advantage allows infected mosquitoes to invade and effectively replace local populations [26]. Further, *w*Mel *Wolbachia* blocks *Aedes*-borne virus transmission [25,27–33], making population replacement a promising long-term control strategy. As implementation of *Wolbachia*-based *Ae. aegypti* replacement programs expand [22,34–36], robust surveillance and detection methods are required to monitor the long-term stability of the infection at intervention sites.

*Aedes aegypti* infected with the *w*Mel *Wolbachia* strain were initially released in Cairns, Australia, where infection frequency has remained high more than 10 years after the initial releases [37], suggesting that these populations are stable. In Medellín, releases of *Wolbachia*-infected *Ae. aegypti* for population replacement occurred between 2017 and 2022 [22]. Although infection frequencies were high in various locations shortly after completion of the program [22], a recent study suggested that the frequency of *Wolbachia*-infected *Ae. aegypti* is substantially lower than previously reported, with estimated individual infection rates ranging from 9.5% to 33.2% in the populations tested [38]. These contrasting findings created uncertainty regarding the long-term persistence of *w*Mel in local populations and highlighted the need for an independent reassessment using complementary sampling and detection approaches.

Monitoring *Wolbachia* infection frequencies following population replacement is essential for assessing long-term stability of the introduced populations. Few studies have evaluated practical approaches for monitoring *Wolbachia* persistence several years after population replacement, when operational funding and surveillance intensity are typically reduced. Most post-release monitoring programs have relied on molecular screening of individual mosquitoes collected from the field using adult trapping methods [39–41], aspirator collections [42] or ovitraps [43]. In some cases, larvae reared from ovitrap collections have been screened to estimate infection frequencies and confirm persistence of the infection [42,43]. Quantitative PCR (qPCR) is commonly used because it provides sensitive and accurate estimates of *Wolbachia* prevalence [22,39,44]. However, processing large numbers of individual specimens is labor-intensive and costly, particularly when surveillance covers large geographic areas and must be sustained for extended periods. Consequently, there is a need to evaluate alternative surveillance strategies that reduce processing costs while still providing reliable information on the persistence and distribution of *Wolbachia* in field populations. Some approaches used by local control programs, such as the use of pooled samples and ovitrap-based collections, can substantially reduce processing time and costs, but may reduce the precision of infection frequency estimates. Evaluating the performance of these approaches is therefore important for developing sustainable surveillance frameworks for *Wolbachia*-based vector control programs.

The aims of this study were (i) to evaluate practical approaches for long-term *Wolbachia* surveillance and (ii) assess the current prevalence of *w*Mel *Wolbachia* in *Ae. aegypti* populations in the selected comunas several years after completion of population replacement. We surveyed mosquito populations using ovitrap collections and field sampling of adult mosquitoes and assessed *Wolbachia* infection using conventional PCR and quantitative PCR (qPCR) in pooled and individual samples. This design allowed us to compare *Wolbachia* detection across sampling strategies (ovitrap-derived versus field-captured adults), sample types (pooled versus individual DNA), and molecular detection methods. We found that *Wolbachia*-infected *Ae. aegypti* remain broadly distributed across the surveyed areas, with infection frequencies generally ranging from approximately 60% to near fixation. Our results further indicate that ovitrap-based sampling combined with conventional PCR provides a cost-effective approach for confirming persistence across large geographical areas, whereas individual qPCR screening provides more precise estimates of infection prevalence.

## Materials and methods

### Ethics statement

At each site, a field technician explained the aims and procedures of the study and requested permission for ovitrap placement and subsequent egg and/or adult mosquito collection. Written informed consent was obtained from the head of the household prior to trap placement. The study protocol was approved by the Ethics Committee of the Instituto Colombiano de Medicina Tropical.

### Study area

Medellín is located in the northwestern part of Colombia, within the Aburrá Valley of the Central Andes (6.2442° N, 75.5812° W), at approximately 1,500 m above sea level. The city is surrounded by mountains and is classified as a tropical rainforest climate under the Köppen–Geiger system [45], a climate characterized by high humidity and relatively stable, mild temperatures throughout the year, with mean annual temperatures typically ranging between 18 °C and 22 °C. Precipitation is abundant and occurs year-round but exhibits a bimodal pattern with peaks during April–June and September–November. Mean annual rainfall is estimated by Sistema de Alerta Temprana de Medellín y el Valle de Aburrá (SIATA) at approximately 2,400 – 3,000 mm.

**Fig 1:**
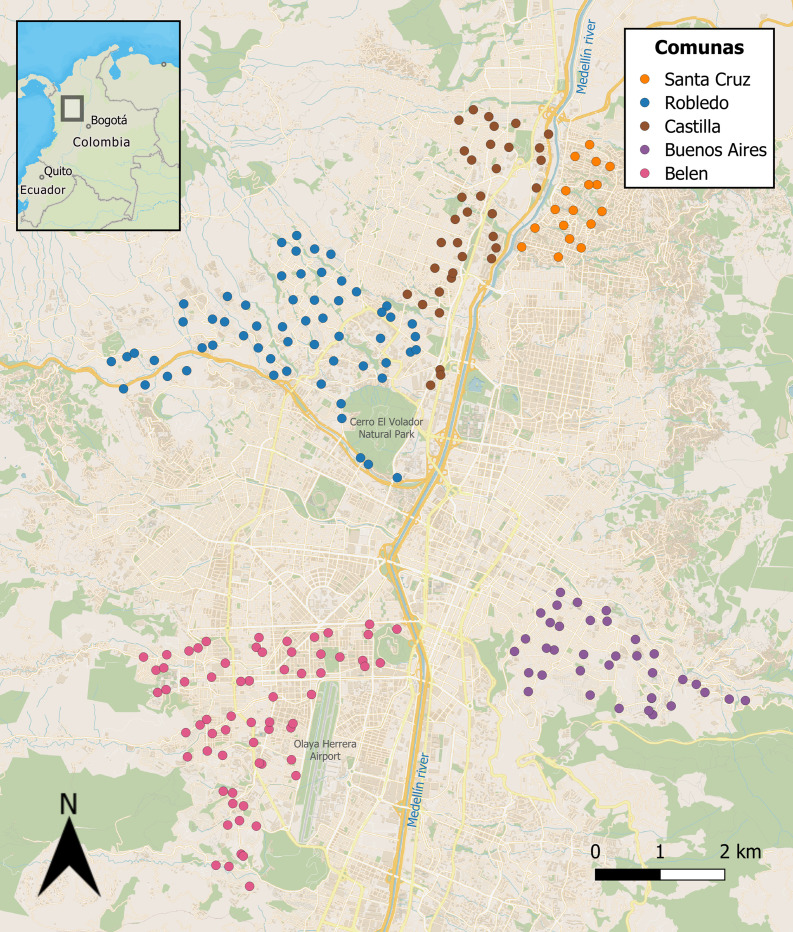
Map of Medellín showing the spatial distribution of sampling locations across the five comunas included in this study. Dots represent the locations where ovitraps were deployed to collect *Aedes aegypti*. Colors indicate neighborhoods where monitoring was conducted. The map was created using OpenStreetMap (https://www.openstreetmap.org) under the Open Database License (https://www.openstreetmap.org/copyright).

A total of 216 ovitraps were deployed across five comunas in Medellín, Colombia: Santa Cruz, Castilla, Robledo, Belén, and Buenos Aires (Fig 1), covering an approximate area of 32.66 km², representing ~29% of Medellin’s urban area (116 km^2^). The area sampled within each comuna was as follows: Santa Cruz (2.20 km²), Castilla (6.10 km²), Robledo (9.46 km²), Belén (8.85 km²), and Buenos Aires (6.05 km²). These comunas comprise an estimated population of approximately 850,000 inhabitants: 121,265 in Santa Cruz, 135,664 in Castilla, 203,600 in Robledo, 221,197 in Belén, and 168,191 in Buenos Aires.

Each comuna was divided into 350 m x 350 m grids, and ovitraps were placed within households when available. Selected comunas were chosen to represent a range of *Wolbachia* infection frequencies [38]: Santa Cruz, Robledo, and Castilla were selected because a recent survey reported infection frequencies of 11.1%, 20.0%, and 54.5%, respectively [38], lower than those observed following release interventions (78.4%, 51.4%, and 100%, respectively) [22]. Belén and Buenos Aires were also included because infection frequencies were relatively similar between studies with frequencies of 32.5% to 38.8% reported in Belen and 62.5 to 86.6 reported in Buenos Aires [22,38]. These comparisons were used to identify comunas spanning a range of reported infection frequencies and were not intended as direct estimates of temporal change, as the underlying studies differed in sampling design and analytical approaches

### Mosquito samples

Mosquitoes from each sampling location were obtained from ovitraps, and adult mosquitoes were collected using Prokopack aspirators [46]. Traps were deployed and mosquitoes collected between August 4 and September 11, 2025, over six sampling cycles. Ovitraps consisted of black plastic containers (~2 L capacity) filled approximately one-third with water. Each ovitrap contained a partially submerged wooden paddle that served as an oviposition substrate. Ovitraps were placed inside households, including living rooms, and other indoor areas, as well as covered semi-outdoor areas, including patios. Wooden paddles and water were replaced weekly throughout the collection period. After retrieval, wooden paddles that contained mosquito eggs were removed, allowed to partially dry, and placed in plastic bags within a humidified container for transport to the laboratory. In the laboratory, eggs on each paddle were examined and counted using a ZEISS Stemi 508 stereo microscope (ZEISS, Oberkochen, Germany). Four to six days after collection, paddles were submerged in water to allow egg eclosion. Larvae were raised in separate containers for each individual ovitrap paddle through emergence and were collected as adults 1 and 4 days after eclosion, identified to species and stored at -20°C in sealed microcentrifuge tubes labeled with collection location and adult age.

Adult mosquitoes were collected indoors at sampling locations using standard Prokopack aspirators. During visits to households with positive ovitraps or visible adult mosquitoes, a trained field technician conducted 10-minute aspiration sessions per house. Collections were performed in all rooms—on walls and surfaces, under beds, tables and other furniture—progressing systematically from the rear to the front of the house. Collected adults were transferred to labeled containers. At the end of each field day, specimens were stored at −20 °C in microcentrifuge tubes sealed with parafilm. In the laboratory, mosquitoes were identified to species [47], sexed, and females that had taken a blood meal were recorded. Households positive for both ovitrap collections and adult captures were removed from subsequent sampling routes. However, we continued to visit households with only a positive ovitrap or adult collection for the remaining sampling cycles.

Mosquitoes obtained from ovitrap collections were used to estimate *Wolbachia* infection frequencies across the study area and to evaluate the utility of pooled-sample screening as a cost-effective surveillance approach. Field-captured adult mosquitoes were used to estimate *Wolbachia* prevalence in the same areas, to compare the performance of conventional PCR and qPCR for *Wolbachia* detection and determine whether infection frequencies inferred from ovitrap-derived samples were consistent with those observed in field populations.

### DNA extraction and PCR

DNA was isolated from individual mosquitoes (1 and 4-day-old adults that emerged from ovitraps or field collected specimens) homogenized in 25 µL of STE buffer (100 mM NaCl, 10 mM Tris-HCl, 1 mM EDTA; pH 8.0) containing 0.25 µL of proteinase K (Shanghai ZJ Bio-Tech Co., Ltd). Samples were incubated at 56 °C for 60 min, followed by enzyme inactivation at 95 °C for 15 min. Extracted DNA was stored at −20 °C until further analysis.

Conventional PCR screening for *Wolbachia* infection was performed as previously described [48] using either pools of a maximum of five individuals obtained from ovitrap collections per household from the same collection date, or single individuals (ovitrap-derived or field-collected adults). For pooled samples, 1 µL of DNA from each individual was combined and diluted in STE buffer in a final volume of 100 µL, of which 1 µL was used as the PCR template. For field-collected females that appeared to have blood-fed, an additional 10X dilution was performed to reduce potential PCR inhibitors associated with blood. Additional dilutions were also performed for females that initially tested negative to ensure that PCR inhibition was not affecting amplification.

*Wolbachia* infection status was determined using primers targeting the IS5 repeat element (IS5F: 5′-GTA TCC AAC AGA TCT AAG C-3′; IS5R: 5′-ATA ACC CTA CTC ATA GCT AG-3′) [31]. When mosquitoes tested negative for *Wolbachia*, *Ae. aegypti*-specific primers (aegF: 5′-CTC TGC GTT GGA TGA ATG AT-3′; aegR: 5′-ATA GCG TGG TAG CCG TAT G-3′) [49] were used as an internal control to confirm that the DNA extracts did not contain PCR inhibitors. Positive and negative controls for both DNA extraction and *Wolbachia* infection were included in all assays; positive controls consisted of *Ae. aegypti* from our laboratory colony infected with *Wolbachia* [48], whereas negative controls included DNA from an uninfected mosquito (for DNA extraction and PCR to detect *Wolbachia*). PCR reactions without template were also included as negative controls. PCR products were visualized by electrophoresis on 1% agarose gels stained with HydraGreen (ACTGene, Inc., Piscataway, USA).

Quantitative real-time PCR (qPCR) was performed as in [22] on adult mosquitoes collected with Prokopack aspirators. TaqMan assays were used to amplify the *Wolbachia* surface protein gene (*wsp*) (wspTM2_FW: 5′-CAT TGG TGT TGG TGT TGG TG-3′; wspTM2_RV: 5′-ACA CCA GCT TTT ACT TGA CCA G-3′; wspTM_probe: LC640-TCC TTT GGA ACC CGC TGT GAA TGA-IowaBlack) [22]. The *Ae. aegypti* ribosomal protein S17 gene (*RPS17*) served as an internal control (Rps17_FW: 5′-TCC GTG GTA TCT CCA TCA AGC T-3′; Rps17_RV: 5′-CAC TTC CGG CAC GTA GTT GTC-3′; Rps17_probe: FAM-CAG GAG GAG GAA CGT GAG CGC AG-BHQ1) [22]. The Rockefeller strain was used as a *Wolbachia*-negative control for qPCR assays. No-template controls were included in each qPCR plate to monitor contamination. A *Wolbachia*-positive *Ae. aegypti* strain [48] was used to confirm assay performance. Relative *Wolbachia* density was estimated from qPCR data as ΔCp (Cpwsp − CpRps17). *Wolbachia*-negative samples did not generate a detectable *wsp* crossing point (Cp) value.

### Statistical analysis

Individual *Wolbachia* infection probabilities were estimated from pooled PCR data. Pool sizes varied depending on mosquito emergence from ovitraps. Therefore, analyses incorporated pool-specific sizes. Bayesian estimates of individual infection probability were obtained assuming a Beta(1,1) prior (uniform prior between 0 and 1) and a pooled likelihood model, in which the probability of a pool testing positive was defined as P(positive)=1−(1 − *p*)^*n*^ where *p* represents individual infection probability and *n* the pool size. Posterior means and 95% credible intervals (CrI) are reported. Differences in infection probability among comunas were evaluated by comparing posterior estimates and their associated 95% credible intervals. Agreement between *Wolbachia* detection in adults obtained by conventional PCR and qPCR was evaluated using McNemar’s test for paired proportions. Analyses were conducted separately for each comuna, with statistical significance defined as *P* < 0.05.

*Wolbachia* infection status was analyzed using generalized linear models (GLMs) with a binomial error distribution and logit link, fitted to raw counts of positive and negative mosquitoes. Detection probability was modeled as a function of PCR method, sex, comuna, and PCR method × sex interaction. Model significance was assessed using analysis of deviance. Because overdispersion was detected, final models were fitted with a quasibinomial error distribution and statistical inference was based on F-tests. Percentages shown in figures are for visualization only. *Wolbachia* prevalence for each site was estimated as total number of *Ae*. *aegypti* that tested positive for *Wolbachia* divided by the total number tested. Association between ovitrap classification and 1-day-old individual infection status was assessed using Fisher’s exact test.

Relative *Wolbachia* density was assessed by qPCR using the *Wolbachia* surface protein gene (*wsp*) normalized against the *Ae. aegypti* reference gene *Rps17*. Density was estimated from ΔCp values, calculated as the difference between the Cp values of *wsp* and *Rps17*. To assess *Wolbachia* density differences, two comparisons were performed: (i) qPCR-positive mosquitoes that tested either positive or negative by conventional PCR, and (ii) field-collected adults versus adults that emerged from ovitrap collections. Normality was evaluated using Shapiro–Wilk tests. Differences in ΔCp values between qPCR + /conventional PCR− and qPCR + /conventional PCR+ mosquitoes were assessed using Welch’s two-sample t-test, whereas differences between field-collected adults and ovitrap-derived adults were assessed using a Wilcoxon rank-sum test. Effect sizes were estimated using Cohen’s d for the Welch’s t-test and the rank-biserial correlation coefficient (r) for the Wilcoxon test. Relative *Wolbachia* density (2^-ΔCp) was calculated for graphical visualization.

To test for the presence of *Wolbachia* prevalence clusters across *Ae*. *aegypti* populations in the sampled comunas, spatial clustering analyses were conducted on the proportion of adults that tested negative for *Wolbachia* infection. Areas of lower proportion of infected *Ae. aegypti* individuals than expected under spatial randomness (i.e., negative-tested samples uniformly distributed across comunas) were detected using spatial scan statistics [50]. Scan statistics are widely used for spatial cluster detection in epidemiology [51–54]. Briefly, the scan procedure searches the study region with a circular window to detect clusters, which are sequentially tested for significance by likelihood ratio after detection. Circular areas with clusters significantly different from randomness (*p* < 0.05), which is derived from 999 Monte Carlo simulations, are designated as clusters. Two separate analyses were conducted, one for adults that emerged from eggs collected in ovitraps and another for adults collected in aspirators, both using a Bernoulli model [50].

Statistical analyses and graphical visualizations were conducted in R version 4.4.2 (R Foundation for Statistical Computing, Vienna, Austria) using RStudio version 2026.01.0 + 392. Maps used to create Figs 1 and 3 were obtained using OpenStreetMap (https://www.openstreetmap.org) under the Open Database License. Spatial clustering analysis was conducted in SaTScan software version 10.3.3, and maps and spatial visualizations were produced in QGIS version 3.44 [55].

## Results

Of the 216 ovitraps deployed in this study, 45.4% of households were positive for eggs (98/216). Further, adult *Ae. aegypti* were collected in 74.3% (81/109) of aspirated households (Table 1). Sixty-five households had both ovitraps positive for eggs and adult collections (S1 File).

**Table 1:** *Wolbachia* detection in *Aedes aegypti* from ovitrap-derived mosquitoes and field-captured adults across the five comunas surveyed. For ovitraps, the number of pools tested, pool positivity (%), posterior mean infection probability (p), and 95% credible intervals (CrI) are shown. Infection probabilities were estimated using a Bayesian pooled infection model incorporating pool size. For field-captured adults, the total number of mosquitoes tested and the numbers of *Wolbachia*-positive and -negative individuals detected by conventional PCR and qPCR are presented.

		Santa Cruz	Castilla	Robledo	Belen	Buenos Aires
**Ovitraps**	Pools tested	15	28	22	24	9
	Pool Positivity (%)	100.0	100.0	77.3	91.7	88.9
	Posterior Mean Infection Probability (p)	0.994	0.996	0.287	0.372	0.768
	95% CrI	0.945–1.000	0.963–1.000	0.186–0.406	0.255–0.502	0.523–0.944
**Adults**	Total mosquitoes	46	72	52	25	14
	Conventional PCR - Positive	21 (45.7%)	34 (47.2%)	23 (44.2%)	16 (64.0%)	10 (71.4%)
	qPCR – Positive	41 (89.1%)	67 (93.1%)	31 (59.6%)	18 (72.0%)	12 (85.7%)

### *Wolbachia* infection in *Aedes aegypti* emerging from ovitraps

Using conventional PCR, *Wolbachia* DNA was detected in mosquito pools from ovitraps collected in all five comunas sampled in Medellín (S1 File). Pool positivity ranged from 77.3% to 100%. Pools derived from Santa Cruz and Castilla had 100% positivity, whereas Robledo exhibited a lower positivity rate (77.3%) (Table 1). Individuals from negative pools in Robledo, Belén and Buenos Aires were subsequently retested by qPCR to confirm the absence of infection and rule out false negatives caused by low bacterial density. All mosquitoes that tested negative for *Wolbachia* by conventional PCR were also negative by qPCR.

Estimates of individual infection probability, accounting for variable pool sizes, indicated infection probabilities consistent with near fixation in Santa Cruz (posterior mean *p* = 0.994, 95% CrI: 0.945–1.000) and Castilla (*p* = 0.996, 95% CrI: 0.963–1.000). Substantially lower infection frequencies were estimated for Robledo (*p* = 0.287, 95% CrI: 0.186–0.406) and Belén (*p* = 0.372, 95% CrI: 0.255–0.502). Buenos Aires showed intermediate infection probabilities (*p* = 0.768, 95% CrI: 0.523–0.944), with greater uncertainty likely reflecting the smaller number of pools analyzed (Table 1).

Comparisons of *Wolbachia* infection probabilities indicated clear differences among comunas. Santa Cruz and Castilla had infection probabilities consistently higher than those observed in Robledo and Belén (Table 1), as reflected by the non-overlapping credible intervals. Buenos Aires showed intermediate infection probabilities than Robledo but greater uncertainty when contrasted with Santa Cruz and Castilla (Table 1).

### *Wolbachia* infection in field-captured *Aedes aegypti* adults

**Fig 2:**
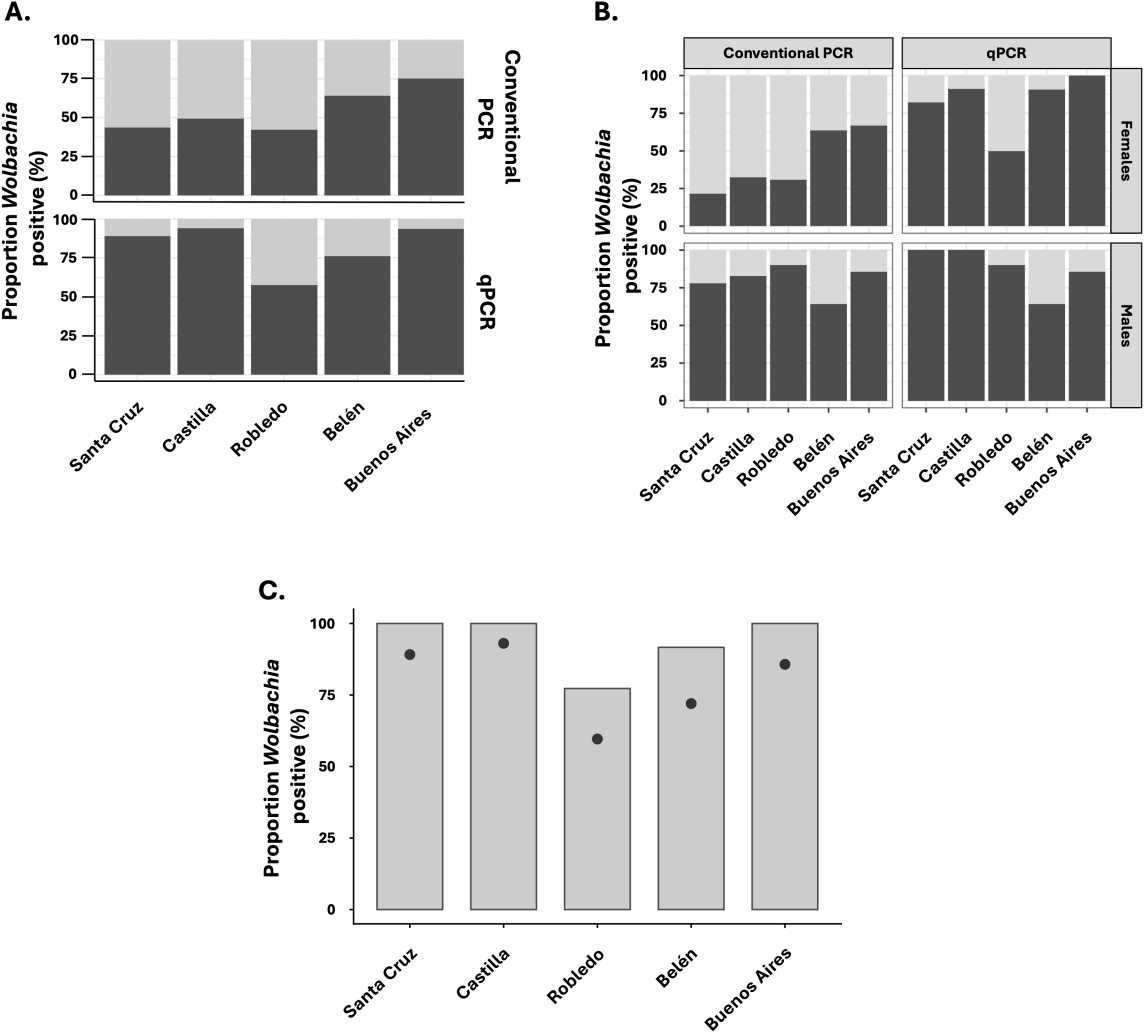
*Wolbachia* detection in field-captured adult *Aedes aegypti* and adults that emerged from ovitraps in the five comunas surveyed. **(A)** Percentage of *Wolbachia*-positive and -negative adult mosquitoes by PCR method. Bars represent proportions calculated from raw individual counts. **(B)** Percentage of *Wolbachia*-positive and *Wolbachia*-negative adults by comuna, sex, and PCR method. Bars represent proportions calculated from raw individuals counts. **(C)** Comparison of *Wolbachia* positivity in ovitrap-derived mosquitoes and field-captured adults by comuna. Bars represent ovitrap positivity (%; determined by conventional PCR), and points represent positivity (%) of field-captured adults (determined by qPCR).

*Wolbachia* infection was assessed in individual adult *Ae. aegypti* collected from households. Infection status was determined using both conventional PCR and qPCR. In total, 209 adults were screened, including 138 females and 71 males. Of these, 104 were positive and 105 were negative by conventional PCR, while 169 were positive and 40 were negative by qPCR (Table 1). qPCR detected significantly higher *Wolbachia* infection frequencies than conventional PCR in Santa Cruz (exact McNemar’s test *P* < 0.0001), Castilla (exact McNemar’s test *P* < 0.0001), and Robledo (exact McNemar’s test *P* = 0.0078) (Fig 2A). No statistically significant differences between detection methods were observed in Belén (exact McNemar’s test *P* = 0.25) or Buenos Aires (exact McNemar’s test *P* = 0.25) (Fig 2A).

Across all comunas, discordant results were exclusively characterized by samples that were negative by conventional PCR but positive by qPCR, whereas no samples were positive by conventional PCR and negative by qPCR. Given the sensitivity of qPCR, these results suggest that *Wolbachia* densities in some samples may be below detection threshold. We found that *Wolbachia* density differed significantly between mosquitoes detected by both conventional PCR and qPCR (group A) versus those only detected by qPCR (group B; S1 Fig). Mean ΔCp values were significantly lower in group A (-1.81) than in group B (-0.95) (Welch’s t-test: t = -3.32, df = 48.95, *P* = 0.0017), with effect size analysis indicating a large difference between groups (Cohen’s d = 0.84). Overall, among qPCR positive mosquitoes, those positive by conventional PCR had ~ 1.8 higher *Wolbachia* densities than those that tested negative (S1 Fig).

Adult sample sizes varied markedly among comunas, ranging from low numbers in Buenos Aires and Belén to substantially larger collections in Castilla and Santa Cruz (Table 1). Despite these differences in sampling effort, *Wolbachia* frequencies by qPCR detection remained high across comunas despite differences in sample size, as comunas with smaller sample sizes also exhibited high detection frequencies (Table 1).

When comparing *Wolbachia* detection in females and males, we observed a significant effect of PCR method (quasibinomial GLM, F₁,₁₈ = 13.93, *P* = 0.0029), with qPCR detecting a higher proportion of positive mosquitoes than conventional PCR. *Wolbachia* positivity also differed significantly between sexes (quasibinomial GLM, F₁,₁₇ = 12.50, *P* = 0.0041), with males showing higher positivity rates than females (Fig 2B). In contrast, no significant differences in *Wolbachia* detection were observed among comunas (quasibinomial GLM, F_4,13_ = 1.14, *P* = 0.38), and the interaction between PCR method and sex was not significant (quasibinomial GLM, F_1,12_ = 1.01, *P* = 0.33), indicating that the effect of detection method was consistent across sexes (Fig 2B).

In Robledo, Belén and Buenos Aires, a small proportion of ovitraps produced *Wolbachia*-negative offspring (5/59, 2/67 and 1/9 ovitraps, respectively). When adults were collected at these locations, some individuals were also *Wolbachia*-negative. However, in one instance (Robledo), an ovitrap produced *Wolbachia* negative adults while 2 of 3 field-captured adults tested positive by qPCR. In these comunas, detection rates obtained using conventional PCR and qPCR appear similar (Fig 2A). In contrast, in comunas where all ovitraps yielded *Wolbachia*-positive offspring, qPCR tended to detect a higher proportion of positive mosquitoes than conventional PCR (Fig 2A). Despite complete ovitrap positivity in some comunas, none showed 100% *Wolbachia* positivity among field-captured adults (Fig 2C).

### Spatial distribution of *Wolbachia* infected adult *Aedes aegypti*

**Fig 3:**
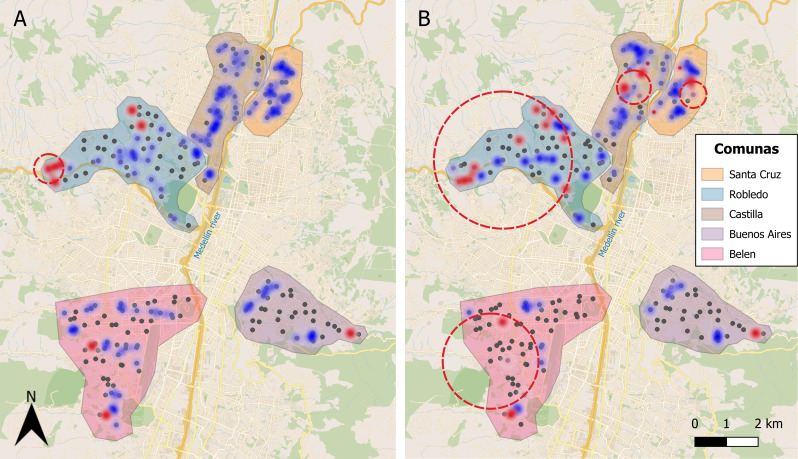
Distribution of *Aedes aegypti* that tested positive (blue) and negative (red) for *Wolbachia* infection and results of spatial clustering analysis. Infection status was determined in adults that emerged from ovitraps **(A)** or proportion of field-captured adults that tested positive or negative **(B)**. Blue or red intensity denotes concentration of *Wolbachia* positive or negative individuals within a 350 m radius of each sampling station (grey dots), and red circles show the clusters of samples that tested negative for *Wolbachia* infection from spatial clustering analysis. Maps were created using OpenStreetMap (https://www.openstreetmap.org) under the Open Database License (https://www.openstreetmap.org/copyright).

The spatial distribution of ovitrap infection status showed that *Wolbachia*-positive traps were widespread across the study area (Fig 3A). In contrast, *Wolbachia*-negative ovitraps were less frequent, and exhibited a more spatially clustered distribution that appeared predominantly at higher elevation sites at the edge of the city limits (one cluster: *p* = 0.06, log likelihood ratio = 8.12) (Fig 3A). Adult collections revealed a higher frequency of *Wolbachia*-negative mosquitoes across multiple comunas, including in Castilla and Santa Cruz, where ovitraps yielded only *Wolbachia*-positive specimens but clusters of *Wolbachia*-negative adults were also detected in all comunas (Belen: *p* = 0.001, log likelihood = 538.72; Robledo: *p* = 0.001, log likelihood = 224.43; Castilla: *p* = 0.001, log likelihood = 245.19; Santa Cruz: *p* = 0.001, log likelihood = 186.66), except for Buenos Aires (Fig 3B). However, *Wolbachia*-negative adults were also recorded in locations where *Wolbachia*-negative ovitraps were observed (Fig 3). *Wolbachia* densities of laboratory-reared adults were higher than field-collected adults in a recent study [56], which may explain some of the differences observed in *Wolbachia* positivity between ovitrap and field collected samples. Therefore, we examined *Wolbachia* levels between the two specimen types and found that ΔCp values significantly differed between field-collected adults and adults from ovitrap collections from the same sites (Wilcoxon rank-sum test, W = 680, *P* = 4.24 × 10^−6^) (S2 Fig). Adults emerging from ovitrap collections exhibited lower ΔCp values (median = −4.27, IQR = 1.44) than field-collected adults (median = −2.63, IQR = 0.798), corresponding to a higher relative *Wolbachia* density (2^-ΔCp^) (S2 Fig). Overall, these findings demonstrate that infection patterns inferred from ovitraps did not consistently predict adult infection status, although negative detection overlapped in some areas.

### Temporal consistency of *Wolbachia* infection status

To evaluate whether mosquito age influenced *Wolbachia* detection, 130 *Ae. aegypti* adults that emerged from ovitraps, 20–30 hours post-eclosion, were screened using conventional PCR. Three individuals were analyzed from each of 38 ovitraps distributed across the comunas (Santa Cruz = 6, Castilla = 10, Robledo = 9, Belén = 10, Buenos Aires = 3); in one ovitrap only two individuals were available for analysis. Overall, 90.2% of individuals tested positive for *Wolbachia*. Eleven individuals tested negative, six of which originated from ovitraps previously classified as *Wolbachia*-negative. The remaining five negative individuals were detected in ovitraps previously classified as *Wolbachia*-positive. Individual *Wolbachia* infection status in 1-day-old adults was strongly associated with infection status of 4-day-old adults (Fisher’s exact test, *P* < 0.0001). All individuals emerging from ovitraps previously classified as *Wolbachia*-negative were themselves negative (0/6), whereas 96.0% (119/124) of individuals from previously classified *Wolbachia*-positive ovitraps were also positive, indicating high concordance between pool-level and individual-level detection.

At several sampling sites, eggs from a subset of ovitraps were collected over two or three consecutive weeks. These repeated samples allowed assessment of short-term temporal consistency in *Wolbachia* detection at these locations. We found that infection status was generally stable over time. Ovitraps initially classified as *Wolbachia*-positive remained positive across sampling weeks. Most ovitraps classified as negative also remained negative; however, one ovitrap (Buenos Aires) that tested negative during the first week was positive in subsequent sampling weeks (S2 File). No other shifts in infection status were observed.

## Discussion

*Aedes aegypti* transinfected with *Wolbachia* is a promising tool for controlling arbovirus transmission. Effective post-release surveillance is essential to evaluate the long-term establishment, persistence, and distribution of *Wolbachia* in target mosquito populations. However, long-term monitoring by local governments may face constraints including limited financial and/or technical resources, making cost-effective and scalable methodologies critical. We sought to evaluate current *Wolbachia* infection frequencies in 5 comunas of Medellín where previous studies reported frequencies ranging from 11.1% to 100% [22,38], and to establish best-practices for post-release *Wolbachia* surveillance in resource-limited settings. We collected *Ae. aegypti* from ovitraps or adults directly from the field and used differing *Wolbachia* detection methods to compare results across sampling and detection methodologies, finding that each provided complementary information. The choice of surveillance methods by control programs should be guided by available resources and existing surveillance infrastructure.

We found that conventional PCR screening of pooled DNA from adults emerging from ovitrap collections is practical and cost-effective for confirming the persistence of *Wolbachia* across large geographical areas. Conventional PCR reliably detected *Wolbachia* in pooled samples; negative testing pools were confirmed by qPCR of individuals, indicating a true absence of infection rather than false negatives due to low *Wolbachia* density. Compared with individual qPCR screening, pooled samples tested by conventional PCR substantially reduces the number of reactions required, reagent consumption, sample processing time, and the need for specialized equipment, making it particularly suitable for routine surveillance in resource-limited settings. However, detection from ovitrap collections depends on appropriate sample handling; eggs should be hatched shortly after collection, as *Wolbachia* density declines with prolonged storage [57,58]. Although less common for *Wolbachia* monitoring, pooled samples are widely used in surveillance programs [59–61], due to reduced processing time and costs when large numbers are analyzed. Importantly, infection frequencies inferred from laboratory-reared adults emerging from ovitrap collections have limited resolution for accurately estimating local infection frequencies, particularly in areas with heterogeneous *Wolbachia* distribution where it may provide an overestimation. Therefore, pooled ovitrap screening is well suited as a first-line surveillance approach for identifying areas where additional monitoring may be warranted.

Individual screening of field-captured adults using qPCR provided a more precise estimate of *Wolbachia* prevalence. Across the comunas assessed, qPCR detected more positive individuals than conventional PCR. The operational significance of these differences remains unclear, as there is currently no universally accepted *Wolbachia* prevalence threshold that triggers additional releases. Although reduced *Wolbachia* densities have been associated with imperfect maternal transmission and diminished pathogen blocking in some experimental studies [62–64], we did not assess these phenotypes in the present study. Nevertheless, the higher sensitivity of qPCR makes it preferable when more accurate estimates of infection frequency are required or when potential declines in *Wolbachia* prevalence need to be investigated. Together, our results support a surveillance strategy in which broad-scale monitoring can be performed using pooled ovitrap samples and conventional PCR followed by qPCR confirmation of negative or ambiguous samples, an approach that retains much of the cost advantage of conventional PCR while improving the accuracy of prevalence estimates.

Despite the differences in detection sensitivity, both methods indicate that *Wolbachia* remains widespread throughout the surveyed areas. This is in contrast to a previous report suggesting substantially lower infection frequencies [38] and highlights the importance of considering differences in sampling design, spatial coverage, and molecular detection methods when comparing post-release surveillance studies. Further, technical caveats regarding PCR assays, such as sample dilutions, thermocycling profiles, and DNA extraction methodology, may potentially affect *Wolbachia* detection capabilities. Although *Wolbachia* was widespread throughout the surveyed comunas, infection frequencies were not homogeneous. Lower frequencies and localized clusters of *Wolbachia*-negative mosquitoes were detected in some areas, indicating that long-term persistence may vary at relatively fine spatial scales. The tendency for some lower-frequency areas to occur near the periphery of the study area is also consistent with the possibility that immigration of uninfected mosquitoes from neighboring municipalities could locally reduce *Wolbachia* frequencies. However, because mosquito movement and immigration were not measured, this interpretation remains speculative.

The factors underlying the observed spatial heterogeneity remain unclear. Differences in mosquito population density, local ecological conditions, environmental factors, mosquito movement from neighboring areas with lower *Wolbachia* frequencies, variation in release history, and potential differences in survival between infected and uninfected mosquitoes under field conditions may all influence the long-term maintenance of *Wolbachia* in field populations. A trend toward clustering of *Wolbachia*-negative ovitraps at higher elevation sites was observed, suggesting that environmental heterogeneity could contribute to these patterns, although environmental variables were not directly measured and these mechanisms remain speculative.

Additionally, the presence of *Wolbachia*-negative adults in areas where ovitrap-derived mosquitoes were entirely positive suggests that while ovitrap-based surveillance generally reflected population infection status, it does not fully capture individual-level variation. Several non-mutually exclusive mechanisms may explain this discrepancy. Imperfect maternal *Wolbachia* transmission can produce uninfected individuals within infected populations [65,66]. Elevated temperatures during development and/or other forms of larval stress may reduce *Wolbachia* density [67–69], affecting male ability to induce CI [62,70] or female ability to transmit the bacteria to their offspring [62]. Infected adults collected directly from the field have lower *Wolbachia* densities than adults from ovitraps reared in the laboratory [56], affecting detection probabilities that we also observed. Vector control activities may also influence the long-term maintenance of *Wolbachia* in field populations [39]. Therefore, long-term surveillance programs should be integrated with broader vector management strategies to ensure that declines in *Wolbachia* frequency are detected early. Identification of persistent low-frequency areas may facilitate targeted investigations into their underlying causes and inform responses, including reinforcement releases, expanded surveillance, or adjustments to complementary vector control activities. Such adaptive management may help maintain stable *Wolbachia* establishment over time.

Interestingly, *Wolbachia* detection frequency observed in field-captured males was greater than in females. Although this pattern could have implications if it reflects consistently lower *Wolbachia* densities in field-derived females, our data do not allow us to distinguish between intrinsic sex-specific differences and variation arising from differences in specimen age, female blood-feeding history, reproductive status, or exposure of field-collected mosquitoes to different environmental conditions. Studies have reported comparable *Wolbachia* densities between lab-reared male and female *Ae. aegypti* in whole bodies [62] and gonadal tissue [71]. Further, *w*Mel densities decline with age in female *Ae. aegypti* [72]. Therefore, additional studies are needed to determine whether the observed sex difference represents a general feature of field populations, and whether it has consequences for maternal transmission or long-term *Wolbachia* persistence.

Overall, our findings demonstrate that *w*Mel *Wolbachia* remains established across the surveyed regions and emphasize the importance of continued surveillance to detect potential localized changes in infection frequency. Importantly, our results show that no single surveillance methodology is optimal for all objectives. Instead, monitoring strategies should be tailored to available resources and specific program goals. For routine long-term surveillance in resource-limited settings, ovitrap collections combined with pooled conventional PCR provide a practical approach for confirming *Wolbachia* persistence and identifying areas that may require additional investigation, whereas individual qPCR remains preferable when accurate estimates of infection prevalence are needed. To aid in these endeavors, we encourage participation of local scientific communities to provide independent evaluations of *Wolbachia*-based release programs. A flexible surveillance framework balances sensitivity, cost, and scalability and may facilitate sustainable long-term monitoring of *Wolbachia*-based vector control programs.

## Supporting information

S1 Fig Comparison of relative *Wolbachia* density (2^-ΔCp) of field captured adults that tested positive by qPCR and conventional PCR (group A) and positive by qPCR but negative by conventional PCR (group B). *Wolbachia* density was estimated by qPCR using ΔCp values. Boxes indicate the interquartile range (IQR), center lines indicate medians, whiskers extend to 1.5 × IQR, and points represent individual mosquitoes.
(TIFF)

S2 Fig Comparison of Relative *Wolbachia* density (2^-ΔCp) in field-collected adult and adults emerging from ovitrap collections. *Wolbachia* density was estimated by qPCR using ΔCp values. Boxes indicate the interquartile range (IQR), center lines indicate medians, whiskers extend to 1.5 × IQR, and points represent individual mosquitoes.
(TIFF)

S1 File Conventional and qPCR results of the mosquitoes assessed in this study collected in each comuna. Coordinates (Lat/Long) indicating the location of each trap are included.
(XLSX)

S2 File Conventional PCR results from individually tested 1-day-old mosquitoes and mosquito pools, with each pool representing mosquitoes collected during a single week at a given site. (XLSX)
