# Supplementary material for: Wolbachia prevalence in Aedes aegypti across five comunas of Medellín, Colombia: Implications for post-release surveillance
Source: PLoS Negl Trop Dis. 2026 Aug 10;20(8):e0014630. doi: 10.1371/journal.pntd.0014630 (PMC13480590; doi:10.1371/journal.pntd.0014630)
Supplement: Supplementary file 1 [file pntd.0014630.s006.docx]

**Reviewer #1

Major comments**

**1. Framing of the manuscript and research objectives is the biggest issue with the manuscript. The introduction and abstract frame the need to assess the Wolbachia infection status amongst Ae. aegypti in Medellín well. However, the issue of surveillance approach and methodology is not framed well. It would be useful to provide an overview of the approaches that have been utilized successfully by groups that have performed past Wolbachia interventions and monitoring, and to explicitly state why alternatives to those methodologies need to be examined.**

We added the following to the introduction (Lines 108-125) that we hope better frames our objectives and previous approaches:

Monitoring *Wolbachia* infection frequencies following population replacement is essential for assessing long-term stability of the introduced populations. Few studies have evaluated practical approaches for monitoring *Wolbachia* persistence several years after population replacement, when operational funding and surveillance intensity are typically reduced. Most post-release monitoring programs have relied on molecular screening of individual mosquitoes collected from the field using adult trapping methods [39–41], aspirator collections [42] or ovitraps [43]. In some cases, larvae reared from ovitrap collections have been screened to estimate infection frequencies and confirm persistence of the infection [42,43]. Quantitative PCR (qPCR) is commonly used because it provides sensitive and accurate estimates of *Wolbachia* prevalence [22,39,44]. However, processing large numbers of individual specimens is labor-intensive and costly, particularly when surveillance covers large geographic areas and must be sustained for extended periods. Consequently, there is a need to evaluate alternative surveillance strategies that reduce processing costs while still providing reliable information on the persistence and distribution of *Wolbachia* in field populations. Some approaches used by local control programs, such as the use of pooled samples and ovitrap-based collections, can substantially reduce processing time and costs, but may reduce the precision of infection frequency estimates. Evaluating the performance of these approaches is therefore important for developing sustainable surveillance frameworks for *Wolbachia*-based vector control programs.

Further, we directly state our aims of this study in the revised Introduction (Lines 127-129): The aims of this study were (i) to evaluate practical approaches for long-term *Wolbachia* surveillance and (ii) assess the current prevalence of *w*Mel *Wolbachia* in *Ae. aegypti* populations in the selected comunas several years after completion of population replacement.

2. **A major point of difference that I can see from previous methodology is the inclusion of pooled samples, where previous studies have typically worked at the level of individual mosquitoes during screening. This is an important issue to consider in your interpretation of your data as it links to a trade-off between the accuracy of the estimate of infection frequency and costs of surveillance. As this is an important caveat for your study design, it would be useful to explicitly state how this issue might have impacted your findings and their interpretation – i.e., under- or over-estimates of the “true” population infection rate**

We have added text to the Discussion to address this (Lines 470-473): “However, pooled samples do not provide individual infection status and have limited resolution for accurately estimating local infection frequencies, particularly in areas with heterogeneous *Wolbachia* distribution where it may provide an under- or overestimation of true infection frequencies”.

**3. Posterior estimates of infection appear to be based on conventional PCR screening, which is shown in this manuscript to be less accurate than qPCR-based methods. If these estimates were based on qPCR, it could be argued that they would better reflect the actual infection rates in each comuna as they would exclude many false negatives. At the very least, this issue should be considered as a caveat in the discussion.**

We agree with the reviewer that qPCR is the best technique that more accurately reflects infection prevalence of field populations. We have reorganized the Discussion to more clearly state that although pooled samples are practical and cost-effective for confirming the persistence of *Wolbachia* across large geographical areas, qPCR is best to accurately assess prevalence.

**4. The utility of conventional PCR as a diagnostic approach is perhaps oversold in the manuscript given the observation that its accuracy is lower than qPCR. Perhaps you could consider advocating for a two-step approach where all samples are screened with conventional PCR and then all negatives are screened with qPCR?**

As mentioned in the previous comment, we do mention the limitations of conventional PCR accurately assessing infection frequencies in field populations and suggest conventional PCR is best as “first-line surveillance approach” (Lines 473-475) and advocate for the two-step approach suggested (Lines 483-487).

**Additionally, there are technical caveats relating to the optimization of PCR assays that could have impacted amplification, and could be mentioned in the discussion, such as issues with sample dilutions, thermocycling profiles, and choice of DNA extraction methodology.**

We agree that these caveats are important. We have included more information in the methods regarding our PCRs and the dilutions we used to reduce the potential effects of inhibitors (Lines 224-229, 233-235). We also mention potential issues regarding PCR assays in the Discussion (Lines 493-495).

**Minor comments**

**6. In the methods section, it would be helpful to specify which specimens and approaches were used for which purpose. i.e., ovitrap specimens were screened with PCR and used to estimate infection rates. Aspirator collections were used to compare the efficacy of conventional PCR and quantitative PCR.**

We added (Lines 207-212): “Mosquitoes obtained from ovitrap collections were used to estimate *Wolbachia* infection frequencies across the study area and to evaluate the utility of pooled-sample screening as a cost-effective surveillance approach. Field-captured adult mosquitoes were used to estimate *Wolbachia* prevalence in the same areas, to compare the performance of conventional PCR and qPCR for *Wolbachia* detection and determine whether infection frequencies inferred from ovitrap-derived samples were consistent with those observed in field populations.

**7. It is unclear if the primers and probes used in this project were newly designed, or if they were adopted from a previous study. It would be helpful to include references highlighting their use, if possible.**

We added the citation for the primers used.

**8. Lines 474-475 – “The use of pooled DNA in Wolbachia screening is common in surveillance studies as it reduces processing time and costs when large numbers of samples are analyzed.” – this statement needs supporting references.**

The reviewer is correct, and we intended to speak more directly about mosquito surveillance in general rather than *Wolbachia* monitoring specifically. Therefore, we changed this to “Although less common for *Wolbachia* monitoring, pooled samples are widely used in surveillance programs” (Line 468-470).

**9. Provide more specific details about past releases and surveillance, rather than using arbitrary terms like low, intermediate, or high to describe Wolbachia infection frequencies. This will enable your readers to more effectively put your work into the context of the literature.**

We have given specific details of the results of the two previous analyses of *Wolbachia*-infected *Ae aegypti* coverage in the revised Introduction (Lines 163-169).

**10. Fig. 2A/B – the y-axis titles do not reflect the data presented. I recommend changing them to ‘Wolbachia-infection rate (%)’, or something similar. There are also no x-axis titles on these panels (panels A, B, and C), and no axes lines on panel A.**

We have amended Figure 2.


**11. Line 108, and in the reference list – wMel – w not italicized**

We have italicized the ‘w’ where *w*Mel appears in the text.

**12. Line 254 - aegypti is capitalized**

We have corrected this.

**13. Line 375 – “In contrast, adult collections revealed a higher frequency of Wolbachia-negative mosquitoes across multiple comunas, including in Castilla and Santa Cruz, where ovitraps yielded only Wolbachia-positive specimens but clusters of Wolbachia-negative adults were also detected in all comunas (Belen: p = 0.001, log likelihood = 538.72; Robledo: p = 0.001, log likelihood = 224.43; Castilla: p = 0.001, log likelihood = 245.19; Santa Cruz: p = 0.001, log likelihood = 186.66), except for Buenos Aires (Figure 3B).” – Could this be because the material derived from ovitraps was pooled, which would make it difficult to detect negative samples.**

In this study, individuals from negative pools were verified as negative by qPCR, suggesting they were true negatives samples rather than individuals with low *Wolbachia* densities. Therefore, it is not clear why we observed this. However, we speculate in the Discussion (Lines 500-503) why we may have observed this, suggesting environmental and physiological factors that may explain this discrepancy. Further, we note that screening via ovitraps may over- or underestimate infection frequencies (lines 470-473).

**14. Line 427 – change ‘infections’ to ‘infection’**

We have corrected this.

**15. In the results section, add test names and relevant test statistics to listed p values**

We have added exact the test names and relevant statistics to the listed P values.

**Reviewer #2 


Major comments**

**1. I would like to begin with the final section of the manuscript. In the last paragraph, the authors suggest the importance of identifying the most appropriate methodology for Wolbachia surveillance following field releases, but they do not provide a clear recommendation. Which method should ultimately be preferred? Should surveillance rely on eggs collected from ovitraps, subsequently reared in the laboratory and analyzed in pools? Should both males and females be analyzed, or only females, given their role in maternal transmission of Wolbachia and arboviruses? These points require a more explicit discussion.**

One of our main goals of this study was to identify practical, cost-effective approaches that could be utilized by budget limited localities. Although we found that that pooled samples inflated *Wolbachia* positivity rates across localities, it was sufficient for *Wolbachia* detection across large geographical areas using conventional PCR. However, we understand that more precise prevalence estimates may be needed, in which case we suggest qPCR. To combine both methods, suggesting in the Discussion (Lines 483-487) use of “pooled ovitrap samples and conventional PCR followed by qPCR confirmation of negative or ambiguous samples” to maintain some of the cost advantage of conventional PCR while improving the accuracy of prevalence estimates. We make no suggestion of specimen sex given that males and females reared in the lab have similar *Wolbachia* densities, which we mention on the Discussion (Lines 529-531). We also state in the Discussion (Lines 459-460 and 545-546) that surveillance programs need to balance sensitivity, cost, and scalability, which depend on the resources of the specific program doing the surveillance.

**2. The decision to rear eggs under laboratory conditions until adulthood may have affected Wolbachia density and, consequently, bacterial detection by conventional PCR. It is well established that Wolbachia density in laboratory-reared mosquitoes is significantly higher than in field-collected individuals (Correa-Antonio et al. 2024 doi: 10.3390/v16020214). It would therefore be highly informative if the authors quantified Wolbachia densities through qPCR and compare: (i) positive versus negative samples by conventional PCR, and (ii) mosquitoes reared under laboratory conditions versus adults directly collected in the field.**

We thank the Reviewer for this suggestion. As expected, we found that individuals positive for *Wolbachia* by qPCR but negative for conventional PCR have lower *Wolbachia* densities than those that tested positive by both methods. We have added this to the Results section (Lines 365-373) as well as added supplemental Figure 1. We also assessed *Wolbachia* densities in a subset of individuals collected from the field and adults that emerged for ovitraps at the same sites (N = 29 each group) and found that adults that emerge from the ovitraps have higher *Wolbachia* densities than field collected individuals. This has also been added to the Results section (Lines 413-422) as well as supplemental Figure2.

3. **The authors emphasize a major discrepancy between the findings of Calle-Tobon et al. (2024) and the present study. However, when comparing both studies, some methodological differences likely contributed to the distinct outcomes. If I understood correctly (otherwise, please clarify in the Methods), pools in the present study consisted of adults originating from the same household, whereas Calle-Tobon et al. pooled specimens collected from different ovitraps to obtain a broader regional overview. Another important difference is that the present study analyzed mosquitoes of both sexes, whereas the previous work included only females.**

The reviewer is correct, in our study pools were formed by individuals from the same household and from the same date, which we state in the methods (Lines 222-224), while in Calle-Tobon et. al. “Pools of Ae. aegypti females were created from each positive ovitrap, with each pool containing 1 to 5 mosquitoes from different epidemiological weeks to avoid overrepresentation of the same breeding source.” We should note that while the previous study suggests they “incubated eggs for 5 days” before hatching, they also state that they used individuals from “different epidemiological weeks”, which suggests 1) that this may not always have been the case, 2) that they used mosquitoes of vastly different ages, or that the adults were stored for long periods. Which of these may pertain to the previous study is not made clear in their methods.

**Indeed, when comparing only conventional PCR results from female mosquitoes, both studies reported remarkably similar findings. In Santa Cruz, Calle-Tobon et al. observed infection frequencies of 11%, 55%, 20%, and 63%, while the authors here found approximately 23%, 30%, 25%, and 60% Wolbachia-positive females in Santa Cruz, Castilla, Robledo, and Buenos Aires, respectively (Figure 2B). A discrepancy was observed only in Belén, where the previous study detected 39% positivity, whereas the present study found nearly 60% infected females. Assuming that conventional PCR sensitivity is linearly associated with Wolbachia density, the consequences of the unbalanced proportions of highly infected males and females warrant much deeper investigation (lines 497–499). This finding is sufficiently important that it should also be highlighted in the Abstract. If this interpretation is correct, the lower proportion of infected females could compromise the self-sustaining population replacement strategy, given that Wolbachia is maternally inherited, and also arbovirus protection, since females with less Wolbachia are more prone to become infected by arbovirus and transmit. Furthermore, the discrepancy between conventional PCR and qPCR positivity rates among females in two of the five comunas (Santa Cruz and Castilla) may also indicate lower Wolbachia densities in females of these areas. Since arbovirus blocking is density-dependent, this observation deserves additional attention and discussion.**

We agree that conventional PCR estimates from female mosquitoes are broadly similar between the two studies in several comunas. However, comparisons should be interpreted cautiously because the studies differed in their sampling approaches. Calle-Tobón et al. estimated infection frequencies using mosquitoes derived from ovitrap collections, whereas our estimates were obtained from field-captured adult mosquitoes. Ovitrap-derived and field-collected samples may yield different estimates of *Wolbachia* prevalence due to differences in the subset of the population sampled and potential differences in *Wolbachia* density. Furthermore, in our study, qPCR consistently detected a greater number of infected individuals than conventional PCR, suggesting that prevalence estimates based solely on conventional PCR may underestimate true infection frequency.

However, in the text of the ms we mention some reasons why low *Wolbachia* levels might have been observed such as age effects, physiological status etc. (lines 525-528).

**4. I missed a comparative table and graphical analysis integrating the results obtained previously in 2021/2022 (Velez et al. 2023), during August–October 2023 (Calle-Tobon et al. 2024), and in the present study across the analyzed comunas. Since Calle-Tobon et al. evaluated only females using conventional PCR, the comparative graph could include both the overall conventional PCR prevalence and female-only prevalence from the present study. Such comparisons would facilitate a much richer discussion regarding temporal fluctuations and their possible explanations, especially considering that mosquito releases ceased in 2022. Given the known temperature sensitivity of wMel, it would be highly informative to compare temperature and rainfall conditions during the collection periods (August–October 2023 versus August–September 2025).**

We agree that temporal comparisons among studies are potentially informative. However, direct quantitative comparisons are complicated by substantial differences in sampling design, diagnostic methodology, and spatial coverage among studies. Vélez et al. relied primarily on individual qPCR screening during the release phase, Calle-Tobón et al. used pooled ovitrap-derived females screened by conventional PCR, whereas the present study included both ovitrap-derived and field-captured adults screened using conventional PCR and qPCR. In addition, sampling intensity and site coverage differed substantially among studies within each comuna. Consequently, prevalence estimates are not directly comparable, and we were concerned that a formal comparative analysis could lead to over-interpretation of methodological rather than biological differences.

However, we do note the following to consider the differences observed between studies (Lines 490-493): “This contrasts a previous report suggesting substantially lower infection frequencies [38] and highlights the importance of considering differences in sampling design, spatial coverage, and molecular detection methods when comparing post-release surveillance studies.”

**5. I also missed a broader discussion of integrated vector management. Since wMel is sensitive to elevated temperatures and Wolbachia-infected mosquitoes tend to maintain smaller egg banks, prolonged heat periods or the use of larvicides/insecticides may disproportionately affect Wolbachia-infected populations (plesase consider Ulrich et al. 2016; Ross et al. 2020; Maciel-de-Freitas et al. 2024; Pavan et al. 2026). What mitigation strategies should be implemented under these scenarios? This issue deserves a broader and more practical discussion.**

We have added to our Discussion information regarding the physiological and environmental factors that can alter *Wolbachia* density levels (Lines 525-528) while also adding discussion about integrated vector management (Lines 517-522).

**6. In some comunas of Medellín, Wolbachia prevalence appears substantially higher regardless of mosquito sex, particularly in Buenos Aires and Belén. Are there plausible ecological or demographic explanations for this pattern, such as lower human density, higher altitude, or smaller native mosquito populations facilitating population replacement? This deserves further exploration.**

We agree that ecological and demographic factors may contribute to the observed differences among comunas. We acknowledge in the Discussion potential influences including mosquito population density, human density, urban structure, altitude, and release history, while noting that future studies will be required to evaluate these hypotheses directly (Lines 500-503).

**7. The authors should better discuss the implications of the higher number of Wolbachia-negative clusters observed in the western and southwestern regions of the city. Could this reflect active or passive migration of native Wolbachia-free vectors into areas without releases? What are the long-term implications? Could increased competition with native populations progressively reduce Wolbachia prevalence over time, particularly considering the lower proportion of infected females observed here?**

We rewrote large portions of the Discussion, and we note that “Lower frequencies and localized clusters of *Wolbachia*-negative mosquitoes were detected in some areas, indicating that long-term persistence may vary at relatively fine spatial scales (Lines 496-498). The following paragraph discusses reasons why this might be the case. We are not sure what the Reviewer means by “increased competition”, but are hesitant to suggest that changes in native populations may reduce infection levels given that more time is needed to determine this as replacement programs are relatively new, and the fact that the original releases in Australia more than a decade ago have remained stable.

**8. The discrepancies among results generated by different sampling strategies and molecular methods need to be much more explicit in the Abstract.**

A major emphasis in our study was to assess approaches that balance accuracy and cost while providing reliable information regarding *Wolbachia* infection persistence for long-term *Wolbachia* surveillance following population replacement which is a need given that more municipalities are implementing this strategy to combat *Aedes* borne diseases. Thus, we feel the discrepancies between the various sampling strategies is best served in the Introduction and Discussion sections.

**9. Finally, the manuscript should emphasize the importance of conducting surveillance analyses such as these in all release sites worldwide, including stronger participation of the broader scientific community to provide independent evaluations of Wolbachia-based release programs.**

We mention the importance of these types of surveillance efforts in various places in the introduction and the Discussion, as specifically encourage participation of local scientific communities in the concluding paragraph of the results (Lines 543-545)

**Minor comments**

**l27–28. Avoid repetitions such as “Aedes aegypti” and “Ae. aegypti”, and “Wolbachia pipientis” and “Wolbachia”.**

We have modified this part to avoid such repetitions.

**l57. Remove “them”.**

We have done this.

**l69. Include “being” before “present throughout...”.**

We have done this.

**l70–71. Consider: “Female Ae. aegypti mosquitoes have a preference...”.**

We have made this change.

**l145–148. In the sentence “Selected comunas were chosen because a previous study [38] reported low infection frequencies in four of these areas compared to levels reported by the WMP following release interventions [22]”, this statement is accurate for three comunas (Santa Cruz, Robledo, and Castilla), but not for Belén, where both studies (Refs. 22 and 38) detected relatively low Wolbachia frequencies (32.5% and 38.8%, respectively).**

You are right, we added in Methods (Lines 163-169), ”Selected comunas were chosen to represent a range of *Wolbachia* infection frequencies [38]: Santa Cruz, Robledo, and Castilla were selected because a recent survey reported infection frequencies of 11.1%, 20.0%, and 54.5%, respectively [38] lower than those observed following release interventions (78.4%, 51.4%, and 100%, respectively) [22]. Belén and Buenos Aires were also included because infection frequencies were relatively similar between studies with frequencies of 32.5% to 38.8% reported in Belen and 62.5 to 86.6 reported in Buenos Aires [22,38].

**l162–163. “Traps were deployed and mosquitoes were collected”.**

We have made this change.

**l186. Please provide a reference for taxonomic identification.**

We added “L.M. Rueda Pictorial keys for the identification of mosquitoes (Diptera: Culicidae) associated with dengue virus transmission Zootaxa, 589 (1) (2004) 1–60-61–60”

**Figure 3. Please include the names of the comunas to facilitate interpretation of the heatmaps.**

We have modified the maps in Figure 3 to outline each comuna in the study.

**l435–436.** **qPCR sensitivity is independent of whether ovitrap-derived or field-collected adults are analyzed.**

The Reviewer is correct. However, to address their previous comment, we assessed *Wolbachia* densities in adults from ovtitraps and those collected in the field from the same sites and found that, as previously reported, significantly higher *Wolbachia* densities were found in ovitrap adults. This could be due to factors related to optimal rearing conditions in the lab and that specimens were of a known age and assessed shortly after emergence. We have added this information to the Results (Lines 363-371 and Supplementary figure 1).

**l507–508. This statement contradicts the discussion presented in lines 476–481. Ovitrap-derived pooled results do not appear to accurately reflect the prevalence observed in individual samples, and “as a result, pooled estimates are associated with greater uncertainty”.**

We have addressed this discrepancy.

**Supplementary files should be entirely written in English.**

We have ensured the supplementary files are entirely in English.

**Reviewer #3**

**The authors could improve the manuscript by better explaining the context of the study: what proportion of the original release area was sampled in this study?**

We mention in the Methods (Lines 154-160) “A total of 216 ovitraps were deployed across five comunas in Medellín, Colombia: Santa Cruz, Castilla, Robledo, Belén, and Buenos Aires (Figure 1), covering an approximate area of 32.66 km², representing ~29% of Medellin’s urban area (116 km^2^). The area sampled within each comuna was as follows: Santa Cruz (2.20 km²), Castilla (6.10 km²), Robledo (9.46 km²), Belén (8.85 km²), and Buenos Aires (6.05 km²).

**How large of an area should be sampled or sub-sampled for long-term surveillance compared to the original release area?**

At present, to our knowledge, there is no standardized recommendation regarding the proportion of a release area that should be sampled during long-term *Wolbachia* surveillance. Sampling strategies are likely to depend on program objectives, available resources, and the degree of spatial heterogeneity expected within the intervention area. Our results suggest that surveillance efforts should prioritize broad geographic coverage and include areas where previous monitoring has identified lower infection frequencies or greater uncertainty regarding *Wolbachia* persistence.

**Why were only certain comunas chosen for sampling in this study and why were they different than the past study they were citing which found different prevalences of Wb infection? Addressing these questions would better support the conclusions related to the first hypothesis that Wb+ prevalence remains high (hard to know if the scope of the sampling relative to the original release area is not put in context).**

We thank the reviewer for this comment. The objective of the present study was not to estimate *Wolbachia* prevalence across the entire Medellín release area, but rather to reassess *Wolbachia* prevalence in selected comunas that had previously shown low, intermediate, or uncertain infection frequencies and to compare alternative surveillance methodologies. We have clarified the rationale for comuna selection in the Methods (Lines 163-169) and now state that the selected comunas were chosen to represent a range of previously reported *Wolbachia* infection frequencies.

**The study did a great job supporting the second hypothesis that conventional PCR on pooled samples can be a useful surveillance approach for long-term monitoring. This hypothesis could be more clearly stated in the introduction but the methods and results clearly showed comparable qualitative results across comunas regardless of method (areas with lower prevalence by conventional PCR were generally also lower by qPCR or conventional PCR on individual samples). The manuscript could improve by clarifying the qPCR methods (specifying the thresholds set by positive and negative controls) and might not need to put the male / female data comparisons in the main text (could move to supplement).**

Positive samples produced characteristic amplification curves with Ct values ranging from 19–28, similar to those observed in the positive controls. Negative samples showed no detectable amplification and therefore no Ct value. This information has been added to the Methods (Line 252-254). We also added more information about the qPCR controls in the methods (250-252).

**The study design looked at 5 comunas in Medellin but it was unclear why those 5 comunas were chosen instead of other areas? The 2024 paper cited by the authors found low prevalence of Wb in comunas that were not sampled in this study -- why was that? Authors should justify why areas that were chosen were chosen and address why some areas may differ in prevalence compared to other areas (i.e. on the border of release sites?).**

We added (Lines 163-169) “Selected comunas were chosen to represent a range of *Wolbachia* infection frequencies [38]: Santa Cruz, Robledo, and Castilla were selected because a recent survey reported infection frequencies of 11.1%, 20.0%, and 54.5%, respectively [38] lower than those observed following release interventions (78.4%, 51.4%, and 100%, respectively) [22]. Belén and Buenos Aires were also included because infection frequencies were relatively similar between studies with frequencies of 32.5% to 38.8% reported in Belen and 62.5 to 86.6 reported in Buenos Aires [22,38]. We have also added to the Discussion possible explanations for why some areas had variable *Wolbachia* coverage.

**More background info is needed to set expectations for which areas might be expected to have lower or higher prevalence and why certain areas were chosen for sampling but not others, particularly why not the areas which were previously reported to have low prevalence in previous work. Please put in context how big the area was that was sampled vs the size of the area that releases were conducted in. Is this area representative of the entire release area? Why or why not.**

As mentioned previously, we have added our rationale for selecting the comunas we assessed and how large the sampling area was compared to the area where releases occurred (Lines 154-160 and 163-169). However, as far as we know, and given that *Wolbachia* releases for population replacement are relatively new, we don’t think it is possible to determine which areas “might be expected to have lower or higher prevalence”. **For the qPCR methods please specify if a negative control was used to set the threshold for positivity. Negative controls were mentioned for conventional PCR but not for qPCR. Were negative and positive controls the same as used for conventional PCR? What thresholds were established from controls to determine positive / negatives in data.**

We have added more information regarding the positive and negative controls used in our PCRs in the Methods (Lines 250-255).

**The hypothesis in the introduction should include what the authors expected from pooled testing vs individual testing (what was the hypothesis for how the different methods compare? and why).**

We state in the Introduction that one of our aims was to determine practical and cost-effective approaches to *Wolbachia* surveillance in resource limited settings. Thus, we wanted to assess the effectiveness of the use of pools tested with conventional PCR to assess *Wolbachia* frequencies and compare those results to testing of field captured adults by qPCR. We suspected that testing of pools might overestimate prevalence estimates which is what we observed. We do not think that this needs to be explicitly stated as it is common knowledge that testing individuals by qPCR would be more accurate, as well as more labor intensive and costly, compared to testing adult pools by PCR.

**Lines 437 - 439 say that the prevalence ranged from 70-100% but it was unclear where these values came from. The results in table 1 show ranges between 59.6% to 93.1% from qPCR of adults and 28.7 - 99.6 for ovitraps, pool positivity between 77 - 100%. Explain where the 70 - 100% comes from (which data source and why different in this line compared to the table) or clarify that the prevalence estimates vary slightly by data source and vary from a lower estimate obtained by ovitraps and individuals adults and a higher estimate from pools (as expected).**

The “70%” was a mistake that regrettably made it into the submitted ms; we thank the reviewer for alerting us of this error. We have changed the text to more accurately reflect our results between PCR methods. We now use 77-100% when speaking of infection frequencies using pools tested by conventional PCR, and ~60-90% when speaking of prevalence estimates derived from qPCR of field captured adults.

**Table 1 could be relabeled to clarify that the group is adults from prokopak collections since all samples were adult samples.**

We have done this.

**There are a few conclusions that need clarification: 1) Line 495, why is it assumed that conventional PCR produced more false negatives. An alternative conclusion could be that qPCR produced more false positives. The controls and thresholds used for positives by qPCR need to be explained in the methods to support which conclusion is correct. 2) Lines 451 - 454, the comunas sampled differed between the two studies. Authors need to explain why and how this could explain some of the results.**

The Reviewer is correct. Based on a comment from R2, we tested this assumption and found that conventional PCR produced more false negatives (Lines 366-371).

Regarding point #2, we were speaking specifically about the assessed comunas. Give that we state “Medellín” in the submitted version, this criticism is valid. Therefore, in the revised version of the ms we have removed text that implies our study suggests coverage of *Wolbachia* infected *Ae. aegypti* is high in Medellín, and we speak specifically to the 5 comunas assessed. This is an important detail, and we are thankful to the Reviewer for pointing this out.

- **Lines 272 - 278 should be in methods section, not results**

We have moved this from the Results to the Methods (Lines 154-160)

**Lines 309 - 316 should be in discussion, not results; Lines 346 - 357 should be in discussion, not results**

For both of these comments, we think that these being included in the Results is fine given that we are discussing our Results. However, we did offer some commentary that should not have been included. Therefore, we deleted speculation regarding interpretation of these results.
