# Supplementary material for: Wolbachia prevalence in Aedes aegypti across five comunas of Medellín, Colombia: Implications for post-release surveillance
Source: PLoS Negl Trop Dis. 2026 Aug 10;20(8):e0014630. doi: 10.1371/journal.pntd.0014630 (PMC13480590; doi:10.1371/journal.pntd.0014630)
Supplement: Supplementary file 2 [file pntd.0014630.s007.docx]

**Reviewer #2:**

**1. The new analyses clearly demonstrate that pooled samples from ovitraps overestimate Wolbachia positivity in the field for at least two reasons: (i) pooling samples increases the probability of detecting at least one infected individual, and (ii) rearing larvae in laboratory to adulthood before screening likely increases Wolbachia density, thereby improving detection. Although these data provide useful complementary information, they should not be interpreted as direct estimates of field infection frequencies.**

We agree that ovitrap-derived laboratory-reared mosquitoes should not be interpreted as providing direct estimates of Wolbachia prevalence in field populations. This distinction was one of the primary motivations for comparing ovitrap-derived and field-collected mosquitoes in the present study. As discussed in the revised manuscript, pooled ovitrap screening is intended as a practical method for confirming Wolbachia persistence over broad geographic areas, particularly if ovitrap collections are already part of municipal mosquito surveillance, as is the case in Medellín. In contrast, individual qPCR screening of field-collected adults provides more accurate estimates of infection prevalence. We also discuss that adults emerging from ovitrap collections exhibited higher Wolbachia densities than field-collected adults, which likely contributes to the observed differences in detection frequency.

To further clarify this point, we have revised the Discussion to more explicitly state this (Lines 475-479):

Importantly, infection frequencies inferred from laboratory-reared adults emerging from ovitrap collections have limited resolution for accurately estimating local infection frequencies, particularly in areas with heterogeneous *Wolbachia* distribution where it may provide an overestimation. Therefore, pooled ovitrap screening is well suited as a first-line surveillance approach for identifying areas where additional monitoring may be warranted.

**2. The manuscript reveals an important biological pattern that deserves greater emphasis. While qPCR detects high Wolbachia positivity in field-collected adults, there is a clear sex bias in conventional PCR, with females exhibiting lower detection rates, most likely due to lower Wolbachia densities. This finding has important epidemiological implications because a higher proportion of females with undetectable or low-density Wolbachia may indicate imperfect maternal transmission and potentially reduced arbovirus blocking. These implications should be explicitly discussed, as they raise important epidemiological concerns that warrant further investigation.**

We agree that if female Ae. aegypti consistently maintain lower Wolbachia densities under field conditions, this could have important implications for maternal transmission and arbovirus blocking. However, our study did not quantify Wolbachia density separately in field-captured, adult males and females, nor did we assess maternal transmission or pathogen blocking. Because field-collected mosquitoes were of unknown age and physiological status, we believe it would be premature to attribute the observed sex difference to an intrinsic biological difference or to infer downstream epidemiological consequences.

Nevertheless, we have revised the Discussion to acknowledge these potential implications while emphasizing that additional studies are needed to determine whether this pattern is consistent across field populations and whether it affects Wolbachia persistence or pathogen blocking (Lines 537-545):

Interestingly, Wolbachia detection frequency observed in field-captured males was greater than in females. Although this pattern could have implications if it reflects consistently lower Wolbachia densities in field-derived females, our data do not allow us to distinguish between intrinsic sex-specific differences and variation arising from differences in specimen age, female blood-feeding history, reproductive status, or exposure of field-collected mosquitoes to different environmental conditions. Studies have reported comparable Wolbachia densities between lab-reared male and female Ae. aegypti in whole bodies [67] and gonadal tissue [70]. Further, wMel densities decline with age in female Ae. aegypti [69]. Therefore, additional studies are needed to determine whether the observed sex difference represents a general feature of field populations, and whether it has consequences for maternal transmission or long-term Wolbachia persistence.

**3. In addition, the current wording in the Abstract and throughout the Discussion, stating that Wolbachia positivity in Medellín ranges from 60–100%, may inadvertently mislead readers. It could suggest either that the frequencies reported by Calle-Tobón et al. were underestimated or that Wolbachia frequencies have increased since that study, neither of which is necessarily supported by the present data. When only female mosquitoes are considered using conventional PCR, the interpretation is considerably different. This distinction should be clearly explained to avoid overinterpretation.**

Our intention was not to imply that the frequencies reported by Calle-Tobón et al. were underestimated or that Wolbachia prevalence has increased since that study, but rather to highlight that different surveillance approaches can yield different estimates of infection frequency. To clarify this point, we have revised the Abstract (Lines 35-37), Methods (Lines 168-170) and Discussion (Lines 495-499) to emphasize that the reported infection frequencies are method-dependent and that direct comparisons among studies should be made cautiously because of differences in sampling design and molecular detection methods.

**4. Regarding my previous comment #7, I also believe the manuscript would benefit from a broader discussion on the management implications of areas where Wolbachia frequencies remain low or absent. Such "pockets" may represent barriers to the long-term stability of Wolbachia invasion and deserve consideration in terms of surveillance and possible supplementary release strategies.**

We agree that persistent areas with lower Wolbachia frequencies have important management implications for long-term surveillance programs. We have expanded the Discussion (Lines 531-535) to emphasize that identifying such areas through routine surveillance can facilitate targeted investigations into their underlying causes and inform adaptive management strategies, including reinforcement releases, expanded surveillance, or adjustments to complementary vector control activities where appropriate. Because our study did not assess mosquito movement, invasion dynamics, or the mechanisms underlying these localized patterns, we have avoided attributing them to any specific process (e.g., immigration of uninfected mosquitoes or differential female infection) and instead discuss these as plausible hypotheses requiring further investigation.

**Specific comments**

**5. l55. Correct the frequency with 60%**

We have corrected this

**6. l367. Exclude "only".**

We have corrected this this

**7. l413. Replace "have been shown to" with "were"**

We have done this.

**8. l418-420. The sentence is confusing, since it seems that lab-reared adults had less Wolbachia than field-collected ones, while the opposite is true.**

We have modified this sentence to make the result clear and to better align with the figure cited. The sentence now reads (Lines 419-421), “Adults emerging from ovitrap collections exhibited lower ΔCp values (median = −4.27, IQR = 1.44) than field-collected adults (median = −2.63, IQR = 0.798), **corresponding to a higher relative** Wolbachia **density (2^-^**^Δ^**^Cp^)** (Figure S2).”

**9. l473. Although I agree that pooled samples may provide overestimation, I can't see how they can understimate Wolbachia positivity.**

We have deleted “under- or” in this sentence. It now reads (Lines 475-478), “Importantly, infection frequencies inferred from laboratory-reared adults emerging from ovitrap collections have limited resolution for accurately estimating local infection frequencies, particularly in areas with heterogeneous *Wolbachia* distribution where it may provide an overestimation.”

**10. Lines 477–481. I still believe it is important to emphasize that caution is needed when interpreting mosquitoes that are positive by qPCR but negative by conventional PCR. These individuals likely harbor lower Wolbachia densities, which may be associated with imperfect maternal transmission and reduced arbovirus blocking. This point has important implications for evaluating the long-term success of Wolbachia establishment.**

We have added the following sentence to the Discussion (Lines 485-487): Although reduced Wolbachia densities have been associated with imperfect maternal transmission and diminished pathogen blocking under some experimental studies [62–64], we did not assess these phenotypes in the present study.

**11. Lines 495–498. An additional explanation worth discussing is that ovitraps negative for Wolbachia along the outskirts of the city may reflect the greater difficulty of maintaining Wolbachia-infected populations in areas that experience continuous immigration of uninfected mosquitoes from neighboring municipalities.**

We thank the reviewer for this suggestion. We agree that continual immigration of uninfected mosquitoes from neighboring municipalities represents a plausible explanation for the lower Wolbachia frequencies observed in some peripheral areas. We have incorporated this possibility into the Discussion as a speculative, non-mutually exclusive mechanism rather than attributing the observed spatial patterns directly to it.

The paragraph now reads (Lines 495-508):

Despite the differences in detection sensitivity, both methods indicate that Wolbachia remains widespread throughout the surveyed areas. This is in contrast to a previous report suggesting substantially lower infection frequencies [38] and highlights the importance of considering differences in sampling design, spatial coverage, and molecular detection methods when comparing post-release surveillance studies. Further, technical caveats regarding PCR assays, such as sample dilutions, thermocycling profiles, and DNA extraction methodology, may potentially affect *Wolbachia* detection capabilities. Although *Wolbachia* was widespread throughout the surveyed comunas, infection frequencies were not homogeneous. Lower frequencies and localized clusters of *Wolbachia*-negative mosquitoes were detected in some areas, indicating that long-term persistence may vary at relatively fine spatial scales. **The tendency for some lower-frequency areas to occur near the periphery of the study area is also consistent with the possibility that immigration of uninfected mosquitoes from neighboring municipalities could locally reduce** Wolbachia **frequencies. However, because mosquito movement and immigration were not measured, this interpretation remains speculative.**

**12. Lines 524–533. I respectfully disagree with the authors' interpretation that these findings should be viewed primarily with caution because of potential effects of mosquito age or nutritional status. Regardless of the mechanisms underlying the lower Wolbachia densities, the biological consequence remains the same: a higher proportion of females with undetectable or low-density Wolbachia implies less efficient maternal transmission and potentially weaker population-level arbovirus blocking. Therefore, I believe the implications of this result deserve stronger emphasis rather than greater caution.**

We appreciate the reviewer's perspective and agree that if female mosquitoes consistently maintain lower *Wolbachia* densities in natural populations, this could have important implications for maternal transmission and arbovirus blocking. However, our field-collected mosquitoes were of unknown age and physiological status, and we did not directly measure *Wolbachia* transmission, viral blocking, or sex-specific *Wolbachia* dynamics over time. Therefore, we believe it would be premature to attribute the observed difference to an intrinsic biological sex effect or to infer consequences for population replacement. We have revised the Discussion to clarify that the observed difference may reflect variation in *Wolbachia* density among the sampled mosquitoes rather than a true sex-specific difference in infection prevalence, while noting that further work is needed to determine whether this pattern is consistent across field populations and whether it has consequences for *Wolbachia* persistence or virus blocking.

However, the Reviewer makes a valid point. We have modified this paragraph to address this point (Lines 537-545), which now reads:

Interestingly, Wolbachia detection frequency observed in field-captured males was greater than in females. Although this pattern could have implications if it reflects consistently lower Wolbachia densities in field-derived females, our data do not allow us to distinguish between intrinsic sex-specific differences and variation arising from differences in specimen age, female blood-feeding history, reproductive status, or exposure of field-collected mosquitoes to different environmental conditions. Studies have generally reported comparable Wolbachia densities between lab-reared male and female Ae. aegypti in whole bodies [67] and gonadal tissue [70], and wMel densities decline with age in female Ae. aegypti [69]. Therefore, additional studies are needed to determine whether the observed sex difference represents a general feature of field populations, and whether it has consequences for maternal transmission or long-term Wolbachia persistence.

**13. l753. Correct the uppercase letters in the author names and title.**

We have corrected this

**14. l801. Correct "mosquitoes"**

We have corrected several instances where we misspelt “mosquitoes” in the text.

**Reviewer #3:**

**1. Table 1: could you provide more commentary in the discussion on why the ovitrap prevalence estimates vary from the adult estimates? Does this change if you look at the same houses with both ovitraps and adults collected -- within a house are the estimates similar?**

We thank the reviewer for this helpful suggestion. We have expanded the Discussion to further address the differences between infection estimates derived from ovitrap collections and field-collected adults. Specifically, we discuss that (1) ovitrap collections sample the offspring of ovipositing females, (2) adults emerging in the laboratory likely have higher Wolbachia densities than field-caught adults, (3) pooled samples are inherently more likely to contain at least one infected individual, and (4) qPCR is more sensitive than conventional PCR.

Adult mosquitoes and ovitrap collections overlapped at 65 sampling locations. However, the two sampling approaches generated fundamentally different types of data. Adult collections yielded infection frequencies based on relatively few mosquitoes per location (typically 1–7 individuals), whereas ovitrap collections were analyzed as pooled presence/absence samples. Consequently, the ovitrap data do not provide household-level prevalence estimates that can be directly compared with adult infection frequencies, and the small numbers of adults collected per location preclude meaningful household-level comparisons. We have therefore clarified in the Discussion that ovitrap screening is intended as a complementary surveillance tool for confirming Wolbachia persistence rather than estimating local infection frequencies.

If the reviewer was instead asking about concordance between ovitrap and adult collections at the same sampling locations, we note that 59 of the 65 ovitrap pools (90.8%) were positive for Wolbachia (with individuals from negative pools subsequently confirmed by qPCR). Furthermore, among these 59 ovitrap-positive locations, all field-collected adults were Wolbachia-positive by qPCR in 48 (81.4%) locations. Household-level PCR results are provided in Supplemental File 1.

**2. Line 53: "tested used" -- one of these needs to be deleted**

We have deleted the word “tested”. The sentence now reads (Lines 52-54), “We collected mosquitoes using ovitraps and indoor sampling and used two molecular methods to detect *Wolbachia*.”

**3. Line 56: "show" should be "showed"?**

We have corrected this.

**4. Lines 74 - 76: maybe make the connection here as to why this study may have been done? If there was high dengue transmission in 2024 was the study done to check if Wolbachia had fallen out to explain the surge in cases? Why were there so many dengue cases in 2024 if Wolbachia prevalence remained high? Comment on other reasons?**

We appreciate the reviewer's suggestion. We believe it would be difficult to infer a relationship between dengue incidence and Wolbachia prevalence from our data. Dengue outbreaks are influenced by numerous factors beyond Wolbachia establishment, including viral introductions, climatic conditions, mosquito abundance, population immunity, migration, and characteristics specific to an urban area such as Medellín. The primary motivation for this study was the uncertainty created by contrasting reports of Wolbachia prevalence in Medellín several years after population replacement. To clarify this point, we have revised the Introduction to emphasize that conflicting estimates of Wolbachia prevalence motivated the present study.

The mentioned paragraph now reads (Lines 96-105):

*Aedes aegypti* infected with the *w*Mel *Wolbachia* strain were initially released in Cairns, Australia, where infection frequency has remained high more than 10 years after the initial releases [37], suggesting that these populations are stable. In Medellín, releases of *Wolbachia*-infected *Ae. aegypti* for population replacement occurred between 2017 and 2022 [22]. Although infection frequencies were high in various locations shortly after completion of the program [22], a recent study suggested that the frequency of *Wolbachia*-infected *Ae. aegypti* is substantially lower than previously reported, with estimated individual infection rates ranging from 9.5 % to 33.2 % in the populations tested [38]. These contrasting findings created uncertainty regarding the long-term persistence of *w*Mel in local populations and highlighted the need for an independent reassessment using complementary sampling and detection approaches.

**5. Line 84: rephrase, "typically absent from" should be "not naturally found in"?**

We have made this change.

**Line 102 - 104: Maybe mention here that the additional high dengue numbers in 2024 also led to questions about the prevalence of Wolbachia.**

Please see our response to comment #4.

**6. Line 277: "1-day -old" delete space after day**

We have made this change.

**7. Line 316: It would be useful to also report the overlap -- how many houses were positive for both or either? I see this is done later in 418 - 419 so maybe give overview earlier?**

We have included in the Results section (Lines 316-317) that 65 locations had ovitraps positive for *Aedes aegypti* eggs and adults were collected.

**8. Line 324: "cause" should be "caused"**

We have made this change.

**9. Line 463 - 464: provide estimate on cost savings? i.e how much cheaper would pooled conventional PCR be? Rough estimate would be nice to see. i.e. 10x or 100x savings?**

We feel that providing a quantitative estimate would be misleading because costs vary substantially among countries and laboratories. In particular, the prices of qPCR reagents, conventional PCR reagents, and specialized equipment differ widely depending on local suppliers, import costs, and currency exchange rates. For example, in Colombia, qPCR reagents are often more than twice as expensive as equivalent products in the United States, and access to real-time PCR instrumentation is frequently limited outside research institutions. Consequently, any estimated cost savings would be highly context-dependent and unlikely to be broadly applicable.

Instead, we have retained a qualitative discussion emphasizing that pooled samples tested by conventional PCR reduces reagent use, sample processing time, and equipment requirements, making it a more practical approach for routine surveillance in many resource-limited settings (Lines 467-471):

Compared with individual qPCR screening, pooled samples tested by conventional PCR substantially reduces the number of reactions required, reagent consumption, sample processing time, and the need for specialized equipment, making it particularly suitable for routine surveillance in resource-limited settings.

**10. Line 490: "contrasts" should be "contrasts to"**

We have corrected this sentence. It now reads, “. This is in contrast to a previous report suggesting substantially lower infection frequencies [38]…”.

**11. Lines 515 - 520: what about survival costs of Wb infection causing lower prevalence in adults?**

We agree that differential survival of Wolbachia-infected mosquitoes could contribute to lower infection frequencies in adult populations and represents another plausible explanation for the observed spatial heterogeneity. Because mosquito survival was not measured in the present study, we have incorporated this possibility into the Discussion as one of several non-mutually exclusive mechanisms that may influence long-term Wolbachia persistence in field populations (Lines 510-517):

The factors underlying the observed spatial heterogeneity remain unclear. Differences in mosquito population density, local ecological conditions, environmental factors, mosquito movement from neighboring areas with lower *Wolbachia* frequencies, and variation in release history, and potential differences in survival between infected and uninfected mosquitoes under field conditions may all influence the long-term maintenance of *Wolbachia* in field populations. A trend toward clustering of *Wolbachia*-negative ovitraps at higher elevation sites was observed, suggesting that environmental heterogeneity could contribute to these patterns, although environmental variables were not directly measured and these mechanisms remain speculative.
