# Supplementary material for: Wolbachia prevalence in Aedes aegypti across five comunas of Medellín, Colombia: Implications for post-release surveillance
Source: PLoS Negl Trop Dis. 2026 Aug 10;20(8):e0014630. doi: 10.1371/journal.pntd.0014630 (PMC13480590; doi:10.1371/journal.pntd.0014630)
Supplement: Peer review history

# Peer review: Decision Letter 0

**DOI:** 10.1371/journal.pntd.0014630.r001

**Authors:** Audrey Lenhart, James Crainey

26 May 2026

Response to Reviewers Revised Manuscript with Track Changes Manuscript

Shaden Kamhawi

co-Editor-in-Chief

PLOS Neglected Tropical Diseases

orcid.org/0000-0003-4304-636XX

Paul Brindley

co-Editor-in-Chief

PLOS Neglected Tropical Diseases

orcid.org/0000-0003-1765-0002

**Journal Requirements:**

1) Please ensure that the CRediT author contributions listed for every co-author are completed accurately and in full.

At this stage, the following Authors/Authors require contributions: Luisa Barrientos, Diego Rincon, Frank W. Avila, and Catalina Alfonso-Parra. Please ensure that the full contributions of each author are acknowledged in the "Add/Edit/Remove Authors" section of our submission form.

The list of CRediT author contributions may be found here: https://journals.plos.org/plosntds/s/authorship#loc-author-contributions

2) Please upload all main figures as separate Figure files in .tif or .eps format. For more information about how to convert and format your figure files please see our guidelines:

https://journals.plos.org/plosntds/s/figures

3) We have noticed that you have uploaded Supporting Information files, but you have not included a list of legends. Please add a full list of legends for your Supporting Information files after the references list.

4) Some material included in your submission may be copyrighted. According to PLOS's copyright policy, authors who use figures or other material (e.g., graphics, clipart, maps) from another author or copyright holder must demonstrate or obtain permission to publish this material under the Creative Commons Attribution 4.0 International (CC BY 4.0) License used by PLOS journals. Please closely review the details of PLOS's copyright requirements here: <a data-cke-saved-href="https://journals.plos.org/plosntds/s/licenses-and-copyright" href="https://journals.plos.org/plosntds/s/licenses-and-copyright">PLOS Licenses and Copyright</a>. If you need to request permissions from a copyright holder, you may use the&nbsp;<a data-cke-saved-href="https://storage.googleapis.com/plos-published-prod/c4ff/Content%20Copyright%20Permission%20Form.pdf?X-Goog-Algorithm=GOOG4-RSA-SHA256&X-Goog-Credential=wombat-sa%40plos-prod.iam.gserviceaccount.com%2F20220817%2Fauto%2Fstorage%2Fgoog4_request&X-Goog-Date=20220817T210608Z&X-Goog-Expires=86400&X-Goog-SignedHeaders=host&X-Goog-Signature=cd9dc868773378f7033b9f8cfb91430167d6275df90bce01342a454f36512530b64f24736fbf68c4fd96da74d663b6ee95b3420bded8fb0e833af2390b896b8706643b476d7f532408921b60576d65b60617070900fd2bcd28eec4cd32fefcf007d3655df17fcc91023329a28479ce0d1c5758174eedcc9e3c1788c5f7dd8523f3ddd871e02c998a20f7dc78ec00f313d4b5a78ba2037a6c7ef0a05dc29817da9368065460c8d8446659f35f0458fefabd3101eb7f926e9cb7a0b53e7bbe47aaed3e0ba52f9af9c2b8f6d7d3a8871b205fc4cbe97384ed37c7604fd7eecfedccf49cba12820b2e3b649541cefde3103cd7d72731b3e8b0248439b4cba691ca8a" href="https://storage.googleapis.com/plos-published-prod/c4ff/Content%20Copyright%20Permission%20Form.pdf?X-Goog-Algorithm=GOOG4-RSA-SHA256&X-Goog-Credential=wombat-sa%40plos-prod.iam.gserviceaccount.com%2F20220817%2Fauto%2Fstorage%2Fgoog4_request&X-Goog-Date=20220817T210608Z&X-Goog-Expires=86400&X-Goog-SignedHeaders=host&X-Goog-Signature=cd9dc868773378f7033b9f8cfb91430167d6275df90bce01342a454f36512530b64f24736fbf68c4fd96da74d663b6ee95b3420bded8fb0e833af2390b896b8706643b476d7f532408921b60576d65b60617070900fd2bcd28eec4cd32fefcf007d3655df17fcc91023329a28479ce0d1c5758174eedcc9e3c1788c5f7dd8523f3ddd871e02c998a20f7dc78ec00f313d4b5a78ba2037a6c7ef0a05dc29817da9368065460c8d8446659f35f0458fefabd3101eb7f926e9cb7a0b53e7bbe47aaed3e0ba52f9af9c2b8f6d7d3a8871b205fc4cbe97384ed37c7604fd7eecfedccf49cba12820b2e3b649541cefde3103cd7d72731b3e8b0248439b4cba691ca8a">PLOS Content Copyright Permission&nbsp;Form</a>

Please respond directly to this email and provide any known details concerning your material's license terms and permissions required for reuse, even if you have not yet obtained copyright permissions or are unsure of your material's copyright compatibility. Once you have responded and addressed all other outstanding technical requirements, you may resubmit your manuscript within Editorial Manager.

Potential Copyright Issues:

- Figures 1 and 3: Please (a) provide a direct link to the base layer of the map (i.e., the country or region border shape) and ensure this is also included in the figure legend; and (b) provide a link to the terms of use / license information for the base layer image or shapefile. We cannot publish proprietary or copyrighted maps (e.g. Google Maps, Mapquest) and the terms of use for your map base layer must be compatible with our CC BY 4.0 license.

Note: if you created the map in a software program like R or ArcGIS, please locate and indicate the source of the basemap shapefile onto which data has been plotted.

If your map was obtained from a copyrighted source please amend the figure so that the base map used is from an openly available source. Alternatively, please provide explicit written permission from the copyright holder granting you the right to publish the material under our CC BY 4.0 license.

If you are unsure whether you can use a map or not, please do reach out and we will be able to help you. The following websites are good examples of where you can source open access or public domain maps:

* U.S. Geological Survey (USGS) - All maps are in the public domain. (http://www.usgs.gov)

* PlaniGlobe - All maps are published under a Creative Commons license so please cite u201cPlaniGlobe, http://www.planiglobe.com, CC BY 2.0u201d in the image credit after the caption. (http://www.planiglobe.com/?lang=enl)

* Natural Earth - All maps are public domain. (http://www.naturalearthdata.com/about/terms-of-use/).

5) Please amend your detailed Financial Disclosure statement. This is published with the article. It must therefore be completed in full sentences and contain the exact wording you wish to be published.

- State the initials, alongside each funding source, of each author to receive each grant. For example: "This work was supported by the National Institutes of Health (####### to AM; ###### to CJ) and the National Science Foundation (###### to AM)."

- State what role the funders took in the study. If the funders had no role in your study, please state: "The funders had no role in study design, data collection and analysis, decision to publish, or preparation of the manuscript.".

If you did not receive any funding for this study, please simply state: u201cThe authors received no specific funding for this work.u201d

**Reviewers' comments:**

**Key Review Criteria Required for Acceptance?**

As you describe the new analyses required for acceptance, please consider the following:

**Methods**

-Are the objectives of the study clearly articulated with a clear testable hypothesis stated?

-Is the study design appropriate to address the stated objectives?

-Is the population clearly described and appropriate for the hypothesis being tested?

-Is the sample size sufficient to ensure adequate power to address the hypothesis being tested?

-Were correct statistical analysis used to support conclusions?

-Are there concerns about ethical or regulatory requirements being met?

Reviewer #1: (No Response)

Reviewer #2: See my comments below

Reviewer #3: - Objectives and hypothesis were clearly stated. The study design looked at 5 comunas in Medellin but it was unclear why those 5 comunas were chosen instead of other areas? The 2024 paper cited by the authors found low prevalence of Wb in comunas that were not sampled in this study -- why was that? For example, the lowest in the 2024 paper were El Poblado and San Antonio de Prado. Authors should justify why areas that were chosen were chosen and address why some areas may differ in prevalence compared to other areas (i.e. on the border of release sites?). More background info is needed to set expectations for which areas might be expected to have lower or higher prevalence and why certain areas were chosen for sampling but not others, particularly why not the areas which were previously reported to have low prevalence in previous work. Please put in context how big the area was that was sampled vs the size of the area that releases were conducted in. Is this area representative of the entire release area? Why or why not.

- Statistical analyses seemed appropriate.

- For the qPCR methods please specify if a negative control was used to set the threshold for positivity. Negative controls were mentioned for conventional PCR but not for qPCR. Were negative and positive controls the same as used for conventional PCR? What thresholds were established from controls to determine positive / negatives in data.

- The hypothesis in the introduction should include what the authors expected from pooled testing vs individual testing (what was the hypothesis for how the different methods compare? and why).

**********

**Results**

-Does the analysis presented match the analysis plan?

-Are the results clearly and completely presented?

-Are the figures (Tables, Images) of sufficient quality for clarity?

Reviewer #1: (No Response)

Reviewer #2: See my comments below

Reviewer #3: - Analysis presented matched the analysis plan.

- Lines 437 - 439 say that the prevalence ranged from 70-100% but it was unclear where these values came from. The results in table 1 show ranges between 59.6% to 93.1% from qPCR of adults and 28.7 - 99.6 for ovitraps, pool positivity between 77 - 100%. Explain where the 70 - 100% comes from (which data source and why different in this line compared to the table) or clarify that the prevalence estimates vary slightly by data source and vary from a lower estimate obtained by ovitraps and individuals adults and a higher estimate from pools (as expected).

- Tables and figures are clear. It was nice to see qualitative results match up between all the methods in Figure 2A and 2C.

-Table 1 could be relabeled to clarify that the adult group is adults from prokopak collections since all samples were adult samples.

**********

**Conclusions**

-Are the conclusions supported by the data presented?

-Are the limitations of analysis clearly described?

-Do the authors discuss how these data can be helpful to advance our understanding of the topic under study?

-Is public health relevance addressed?

Reviewer #1: (No Response)

Reviewer #2: See my comments below

Reviewer #3: - In general, conclusions were well supported by the data presented. There are a few conclusions that need clarification: 1) Line 495, why is it assumed that conventional PCR produced more false negatives. An alternative conclusion could be that qPCR produced more false positives. The controls and thresholds used for positives by qPCR need to be explained in the methods to support which conclusion is correct. 2) Lines 451 - 454, the comunas sampled differed between the two studies. Authors need to explain why and how this could explain some of the results.

**********

**Editorial and Data Presentation Modifications?**

Use this section for editorial suggestions as well as relatively minor modifications of existing data that would enhance clarity. If the only modifications needed are minor and/or editorial, you may wish to recommend “Minor Revision” or “Accept”.

Reviewer #1: (No Response)

Reviewer #2: See my comments below

Reviewer #3: - Lines 272 - 278 should be in methods section, not results

- Lines 309 - 316 should be in discussion, not results

- Lines 346 - 357 should be in discussion, not results

**********

**Summary and General Comments**

Use this section to provide overall comments, discuss strengths/weaknesses of the study, novelty, significance, general execution and scholarship. You may also include additional comments for the author, including concerns about dual publication, research ethics, or publication ethics. If requesting major revision, please articulate the new experiments that are needed.

Reviewer #1: In their manuscript, ‘Wolbachia prevalence in Aedes aegypti across five comunas of Medellín, Colombia: implications for post-release surveillance’, the authors assess the current post-release prevalence of Wolbachia in Medellín, Colombia, and also compare the efficacy of conventional PCR and quantitative PCR diagnostics for Wolbachia detection. The authors use a combination of ovitrap and aspirator sampling to collect mosquito material from five comunas. DNA extractions were performed on pooled or individual samples, which were screened for the presence of Wolbachia via one of the two PCR methods. Bayesian estimates of infection rates were made on pooled samples from the ovitraps. The authors found that Wolbachia infection frequency varied by comuna, that qPCR was a more sensitive detection method, and there was a higher detection rate amongst males than females. The manuscript is well written, timely, and has sufficient detail to replicate the assays performed.

Major comments

1. Framing of the manuscript and research objectives is the biggest issue with the manuscript. The introduction and abstract frame the need to assess the Wolbachia infection status amongst Ae. aegypti in Medellín well. However, the issue of surveillance approach and methodology is not framed well. It would be useful to provide an overview of the approaches that have been utilized successfully by groups that have performed past Wolbachia interventions and monitoring, and to explicitly state why alternatives to those methodologies need to be examined.

2. A major point of difference that I can see from previous methodology is the inclusion of pooled samples, where previous studies have typically worked at the level of individual mosquitoes during screening. This is an important issue to consider in your interpretation of your data as it links to a trade-off between the accuracy of the estimate of infection frequency and costs of surveillance.

Lines 476 – 481 – “However, pooled data provide only an estimate of infection frequency at the group level and do not reveal infection status of individuals. As a result, pooled estimates are associated with greater uncertainty, particularly when the number of pools analyzed is limited, highlighting the importance of adequate sampling effort as well as careful interpretation of pooled data when inferring Wolbachia infection frequencies in field populations.” – As this is an important caveat for your study design, it would be useful to explicitly state how this issue might have impacted your findings and their interpretation – i.e., under- or over-estimates of the “true” population infection rate

3. Posterior estimates of infection appear to be based on conventional PCR screening, which is shown in this manuscript to be less accurate than qPCR-based methods. If these estimates were based on qPCR it could be argued that they would better reflect the actual infection rates in each comuna as they would exclude many false negatives. At the very least, this issue should be considered as a caveat in the discussion.

4. The utility of conventional PCR as a diagnostic approach is perhaps oversold in the manuscript given the observation that its accuracy is lower than qPCR. Perhaps you could consider advocating for a two-step approach where all samples are screened with conventional PCR and then all negatives are screened with qPCR? Additionally, there are technical caveats relating to the optimization of PCR assays that could have impacted amplification, and could be mentioned in the discussion, such as issues with sample dilutions, thermocycling profiles, and choice of DNA extraction methodology.

5. The observation of sex-specific differences in Wolbachia detection is interesting but underexplored in the discussion. Wolbachia density is typically higher in female insects given the fact that it is maternally transmitted. For this reason, it would be unlikely that Wolbachia infection frequencies would be higher in males than in females. Given that female mosquitoes are larger, could the issue be that a lower dilution led to a high concentration of PCR inhibitors in the reaction?

Minor comments

6. In the methods section, it would be helpful to specify which specimens and approaches were used for which purpose. i.e., ovitrap specimens were screened with PCR and used to estimate infection rates. Aspirator collections were used to compare the efficacy of conventional PCR and quantitative PCR.

7. It is unclear if the primers and probes used in this project were newly designed, or if they were adopted from a previous study. It would be helpful to include references highlighting their use, if possible.

8. Lines 474-475 – “The use of pooled DNA in Wolbachia screening is common in surveillance studies as it reduces processing time and costs when large numbers of samples are analyzed.” – this statement needs supporting references.

9. Provide more specific details about past releases and surveillance, rather than using arbitrary terms like low, intermediate, or high to describe Wolbachia infection frequencies. This will enable your readers to more effectively put your work into the context of the literature.

10. Fig. 2A/B – the y-axis titles do not reflect the data presented. I recommend changing them to ‘Wolbachia-infection rate (%)’, or something similar. There are also no x-axis titles on these panels (panels A, B, and C), and no axes lines on panel A.

11. Line 108, and in the reference list – wMel – w not italicized

12. Line 254 - aegypti is capitalized

13. Line 375 – “In contrast, adult collections revealed a higher frequency of Wolbachia-negative mosquitoes across multiple comunas, including in Castilla and Santa Cruz, where ovitraps yielded only Wolbachia-positive specimens but clusters of Wolbachia-negative adults were also detected in all comunas (Belen: p = 0.001, log likelihood = 538.72; Robledo: p = 0.001, log likelihood = 224.43; Castilla: p = 0.001, log likelihood = 245.19; Santa Cruz: p = 0.001, log likelihood = 186.66), except for Buenos Aires (Figure 3B).” – Could this be because the material derived from ovitraps was pooled, which would make it difficult to detect negative samples.

14. Line 427 – change ‘infections’ to ‘infection’

15. In the results section, add test names and relevant test statistics to listed p values

Reviewer #2: Barrientos et al. investigated the prevalence of mosquitoes infected with Wolbachia across five comunas in Medellín, Colombia, four years after the releases of Wolbachia-infected Aedes aegypti mosquitoes aimed at replacing arbovirus-susceptible vector populations with more resistant ones. The main motivation for the study was the previous report by Calle-Tobon et al. (2024), which detected low frequencies of wMel-infected mosquitoes in the city, ranging from 9.5% to 33.2%. The authors screened mosquitoes using conventional PCR from eggs collected in ovitraps, reared to adulthood under laboratory conditions, and analyzed in pools (2–5 individuals), as well as by conventional PCR and qPCR from field-collected adults.

The results obtained through the different methodologies varied substantially, clearly demonstrating that Wolbachia prevalence estimates are highly method-dependent. Pool analyses showed the highest prevalence values (77.3–100%), whereas conventional PCR from field-collected adults yielded lower frequencies (44.2–71.4). As expected, qPCR detected a higher proportion of positive field-collected adults (59.6–93.1%). Particularly noteworthy was the discrepancy in positivity rates between field-collected females (~20–70%) and males (~60–85%) using conventional PCR, which may indicate lower Wolbachia densities in field females.

Overall, the manuscript is highly relevant for public health, vector surveillance, and arbovirus transmission studies. The manuscript is generally clear and well written. However, the Discussion still requires a more in-depth interpretation of the findings and a broader contextualization regarding the long-term use of Wolbachia-based strategies for arbovirus control. My major comments are outlined below.

Major comments

1. I would like to begin with the final section of the manuscript. In the last paragraph, the authors suggest the importance of identifying the most appropriate methodology for Wolbachia surveillance following field releases, but they do not provide a clear recommendation. Which method should ultimately be preferred? Should surveillance rely on eggs collected from ovitraps, subsequently reared in the laboratory and analyzed in pools? Should both males and females be analyzed, or only females, given their role in maternal transmission of Wolbachia and arboviruses? These points require a more explicit discussion.Regarding the method selection, it is important to consider that surveillance approaches must be simple, reliable, and preferably cost-effective, allowing vector control programs to identify areas with higher or lower frequencies of Wolbachia-infected mosquitoes. Based on the results presented here, pooled samples clearly inflated Wolbachia positivity rates across localities, consequently reducing the ability to accurately identify areas with lower coverage. On the other hand, methodological sensitivity is also critical to ensure accurate detection. From this perspective, qPCR would appear to be the most suitable method. However, due to its high sensitivity, qPCR will also detect individuals carrying very low Wolbachia densities. The literature has been demonstrating that females with low Wolbachia densities transmit the bacterium less efficiently to their offspring and become more susceptible to arbovirus infection (Martinez et al. 2014; doi: 10.1371/journal.ppat.1004369). In both cases, these areas would likely require reinforcement releases of wMel mosquitoes by surveillance programs. Therefore, from an operational and epidemiological standpoint, I would argue that conventional PCR of individuals from the field may ultimately represent the most informative approach for long-term surveillance. I recommend the authors to provide a in-depth discussion in this sense.

2. The decision to rear eggs under laboratory conditions until adulthood may have affected Wolbachia density and, consequently, bacterial detection by conventional PCR. It is well established that Wolbachia density in laboratory-reared mosquitoes is significantly higher than in field-collected individuals (Correa-Antonio et al. 2024 doi: 10.3390/v16020214). It would therefore be highly informative if the authors quantified Wolbachia densities through qPCR and compare: (i) positive versus negative samples by conventional PCR, and (ii) mosquitoes reared under laboratory conditions versus adults directly collected in the field.

3. The authors emphasize a major discrepancy between the findings of Calle-Tobon et al. (2024) and the present study. However, when comparing both studies, some methodological differences likely contributed to the distinct outcomes. If I understood correctly (otherwise, please clarify in the Methods), pools in the present study consisted of adults originating from the same household, whereas Calle-Tobon et al. pooled specimens collected from different ovitraps to obtain a broader regional overview. Another important difference is that the present study analyzed mosquitoes of both sexes, whereas the previous work included only females. Indeed, when comparing only conventional PCR results from female mosquitoes, both studies reported remarkably similar findings. In Santa Cruz, Calle-Tobon et al. observed infection frequencies of 11%, 55%, 20%, and 63%, while the authors here found approximately 23%, 30%, 25%, and 60% Wolbachia-positive females in Santa Cruz, Castilla, Robledo, and Buenos Aires, respectively (Figure 2B). A discrepancy was observed only in Belén, where the previous study detected 39% positivity, whereas the present study found nearly 60% infected females. Assuming that conventional PCR sensitivity is linearly associated with Wolbachia density, the consequences of the unbalanced proportions of highly infected males and females warrant much deeper investigation (lines 497–499). This finding is sufficiently important that it should also be highlighted in the Abstract. If this interpretation is correct, the lower proportion of infected females could compromise the self-sustaining population replacement strategy, given that Wolbachia is maternally inherited, and also arbovirus protection, since females with less Wolbachia are more prone to become infected by arbovirus and transmit. Furthermore, the discrepancy between conventional PCR and qPCR positivity rates among females in two of the five comunas (Santa Cruz and Castilla) may also indicate lower Wolbachia densities in females of these areas. Since arbovirus blocking is density-dependent, this observation deserves additional attention and discussion.

4. I missed a comparative table and graphical analysis integrating the results obtained previously in 2021/2022 (Velez et al. 2023), during August–October 2023 (Calle-Tobon et al. 2024), and in the present study across the analyzed comunas. Since Calle-Tobon et al. evaluated only females using conventional PCR, the comparative graph could include both the overall conventional PCR prevalence and female-only prevalence from the present study. Such comparisons would facilitate a much richer discussion regarding temporal fluctuations and their possible explanations, especially considering that mosquito releases ceased in 2022. Given the known temperature sensitivity of wMel, it would be highly informative to compare temperature and rainfall conditions during the collection periods (August–October 2023 versus August–September 2025).

5. I also missed a broader discussion of integrated vector management. Since wMel is sensitive to elevated temperatures and Wolbachia-infected mosquitoes tend to maintain smaller egg banks, prolonged heat periods or the use of larvicides/insecticides may disproportionately affect Wolbachia-infected populations (plesase consider Ulrich et al. 2016; Ross et al. 2020; Maciel-de-Freitas et al. 2024; Pavan et al. 2026). What mitigation strategies should be implemented under these scenarios? This issue deserves a broader and more practical discussion.

6. In some comunas of Medellín, Wolbachia prevalence appears substantially higher regardless of mosquito sex, particularly in Buenos Aires and Belén. Are there plausible ecological or demographic explanations for this pattern, such as lower human density, higher altitude, or smaller native mosquito populations facilitating population replacement? This deserves further exploration.

7. The authors should better discuss the implications of the higher number of Wolbachia-negative clusters observed in the western and southwestern regions of the city. Could this reflect active or passive migration of native Wolbachia-free vectors into areas without releases? What are the long-term implications? Could increased competition with native populations progressively reduce Wolbachia prevalence over time, particularly considering the lower proportion of infected females observed here?

8. The discrepancies among results generated by different sampling strategies and molecular methods need to be much more explicit in the Abstract.

9. Finally, the manuscript should emphasize the importance of conducting surveillance analyses such as these in all release sites worldwide, including stronger participation of the broader scientific community to provide independent evaluations of Wolbachia-based release programs.

Minor comments

l27–28. Avoid repetitions such as “Aedes aegypti” and “Ae. aegypti”, and “Wolbachia pipientis” and “Wolbachia”.

l57. Remove “them”.

l69. Include “being” before “present throughout...”.

l70–71. Consider: “Female Ae. aegypti mosquitoes have a preference...”.

l145–148. In the sentence “Selected comunas were chosen because a previous study [38] reported low infection frequencies in four of these areas compared to levels reported by the WMP following release interventions [22]”, this statement is accurate for three comunas (Santa Cruz, Robledo, and Castilla), but not for Belén, where both studies (Refs. 22 and 38) detected relatively low Wolbachia frequencies (32.5% and 38.8%, respectively).

l162–163. “Traps were deployed and mosquitoes were collected”.

l186. Please provide a reference for taxonomic identification.

Figure 3. Please include the names of the comunas to facilitate interpretation of the heatmaps.

l435–436. qPCR sensitivity is independent of whether ovitrap-derived or field-collected adults are analyzed.

l507–508. This statement contradicts the discussion presented in lines 476–481. Ovitrap-derived pooled results do not appear to accurately reflect the prevalence observed in individual samples, and “as a result, pooled estimates are associated with greater uncertainty”.

Supplementary files should be entirely written in English.

Reviewer #3: Overall, the manuscript was well written with two hypotheses 1) Wb prevalence remains high in release areas after releases and 2) pooled ovitrap samples using conventional PCR can be used to monitor prevalence post-release to save costs of more intensive individual testing of adults. The authors could improve the manuscript by better explaining the context of the study: what proportion of the original release area was sampled in this study?, how large of an area should be sampled or sub-sampled for long-term surveillance compared to the original release area?, why were only certain comunas chosen for sampling in this study and why were they different than the past study they were citing which found different prevalences of Wb infection? Addressing these questions would better support the conclusions related to the first hypothesis that Wb+ prevalence remains high (hard to know if the scope of the sampling relative to the original release area is not put in context). The study did a great job supporting the second hypothesis that conventional PCR on pooled samples can be a useful surveillance approach for long-term monitoring. This hypothesis could be more clearly stated in the introduction but the methods and results clearly showed comparable qualitative results across comunas regardless of method (areas with lower prevalence by conventional PCR were generally also lower by qPCR or conventional PCR on individual samples). The manuscript could improve by clarifying the qPCR methods (specifying the thresholds set by positive and negative controls) and might not need to put the male / female data comparisons in the main text (could move to supplement).

**********

PLOS authors have the option to publish the peer review history of their article (what does this mean?). If published, this will include your full peer review and any attached files.

If you choose “no”, your identity will remain anonymous but your review may still be made public.

**Do you want your identity to be public for this peer review?**  For information about this choice, including consent withdrawal, please see our Privacy Policy.

Reviewer #1: **Yes:**  Eric Caragata

Reviewer #2: No

Reviewer #3: No

**Figure resubmission:**

**Reproducibility:** -->To enhance the reproducibility of your results, we recommend that authors of applicable studies deposit laboratory protocols in protocols.io, where a protocol can be assigned its own identifier (DOI) such that it can be cited independently in the future. Additionally, PLOS ONE offers an option to publish peer-reviewed clinical study protocols. Read more information on sharing protocols at https://plos.org/protocols?utm_medium=editorial-email&utm_source=authorletters&utm_campaign=protocols

# Author comment: Author response to Decision Letter 1

**DOI:** 10.1371/journal.pntd.0014630.r002

7 Jul 2026

Attachment Submitted filename: Response to Reviewers_Updated.docx

# Peer review: Decision Letter 1

**DOI:** 10.1371/journal.pntd.0014630.r003

**Authors:** Audrey Lenhart, James Crainey

21 Jul 2026

Wolbachia prevalence in Aedes aegypti across five comunas of Medellín, Colombia: implications for post-release surveillance

Response to Reviewers Revised Manuscript with Track Changes Manuscript

Shaden Kamhawi

co-Editor-in-Chief

PLOS Neglected Tropical Diseases

orcid.org/0000-0003-4304-636XX

Paul Brindley

co-Editor-in-Chief

PLOS Neglected Tropical Diseases

orcid.org/0000-0003-1765-0002

**Additional Editor Comments (if provided):** **Journal Requirements:**

**Reviewers' comments:**

**Key Review Criteria Required for Acceptance?**

As you describe the new analyses required for acceptance, please consider the following:

**Methods**

-Are the objectives of the study clearly articulated with a clear testable hypothesis stated?

-Is the study design appropriate to address the stated objectives?

-Is the population clearly described and appropriate for the hypothesis being tested?

-Is the sample size sufficient to ensure adequate power to address the hypothesis being tested?

-Were correct statistical analysis used to support conclusions?

-Are there concerns about ethical or regulatory requirements being met?

Reviewer #1: (No Response)

Reviewer #2: See my comments below

Reviewer #3: - The methods describing why certain comunas were chosen is much more clear - great job.

**********

**Results**

-Does the analysis presented match the analysis plan?

-Are the results clearly and completely presented?

-Are the figures (Tables, Images) of sufficient quality for clarity?

Reviewer #1: (No Response)

Reviewer #2: See my comments below

Reviewer #3: (No Response)

**********

**Conclusions**

-Are the conclusions supported by the data presented?

-Are the limitations of analysis clearly described?

-Do the authors discuss how these data can be helpful to advance our understanding of the topic under study?

-Is public health relevance addressed?

Reviewer #1: (No Response)

Reviewer #2: See my comments below

Reviewer #3: (No Response)

**********

**Editorial and Data Presentation Modifications?**

Use this section for editorial suggestions as well as relatively minor modifications of existing data that would enhance clarity. If the only modifications needed are minor and/or editorial, you may wish to recommend “Minor Revision” or “Accept”.

Reviewer #1: (No Response)

Reviewer #2: See my comments below

Reviewer #3: - Line 53: "tested used" -- one of these needs to be deleted

Line 56: "show" should be "showed"?

- Lines 74 - 76: maybe make the connection here as to why this study may have been done? If there was high dengue transmission in 2024 was the study done to check if Wolbachia had fallen out to explain the surge in cases? Why were there so many dengue cases in 2024 if Wolbachia prevalence remained high? Comment on other reasons?

- Line 84: rephrase, "typically absent from" should be "not naturally found in"?

- Line 102 - 104: Maybe mention here that the additional high dengue numbers in 2024 also led to questions about the prevalence of Wolbachia.

- Line 277: "1-day -old" delete space after day

- Line 316: It would be useful to also report the overlap -- how many houses were positive for both or either? I see this is done later in 418 - 419 so maybe give overview earlier?

- Line 324: "cause" should be "caused"

- Line 463 - 464: provide estimate on cost savings? i.e how much cheaper would pooled conventional PCR be? Rough estimate would be nice to see. i.e. 10x or 100x savings?

- Line 490: "contrasts" should be "contrasts to"

- Lines 515 - 520: what about survival costs of Wb infection causing lower prevalence in adults?

**********

**Summary and General Comments**

Use this section to provide overall comments, discuss strengths/weaknesses of the study, novelty, significance, general execution and scholarship. You may also include additional comments for the author, including concerns about dual publication, research ethics, or publication ethics. If requesting major revision, please articulate the new experiments that are needed.

Reviewer #1: All of my previous comments have been addressed to my satisfaction.

Reviewer #2: The authors have substantially improved the manuscript and have addressed many of my previous comments. However, I believe that additional revisions are still necessary before the manuscript is suitable for publication, particularly regarding the results arising from my previous comments #2 and #3 (specifically the analyses added after comment #3).

The new analyses clearly demonstrate that pooled samples from ovitraps overestimate Wolbachia positivity in the field for at least two reasons: (i) pooling samples increases the probability of detecting at least one infected individual, and (ii) rearing larvae in laboratory to adulthood before screening likely increases Wolbachia density, thereby improving detection. Although these data provide useful complementary information, they should not be interpreted as direct estimates of field infection frequencies.

The manuscript reveals an important biological pattern that deserves greater emphasis. While qPCR detects high Wolbachia positivity in field-collected adults, there is a clear sex bias in conventional PCR, with females exhibiting lower detection rates, most likely due to lower Wolbachia densities. This finding has important epidemiological implications because a higher proportion of females with undetectable or low-density Wolbachia may indicate imperfect maternal transmission and potentially reduced arbovirus blocking. These implications should be explicitly discussed, as they raise important epidemiological concerns that warrant further investigation.

In addition, the current wording in the Abstract and throughout the Discussion, stating that Wolbachia positivity in Medellín ranges from 60–100%, may inadvertently mislead readers. It could suggest either that the frequencies reported by Calle-Tobón et al. were underestimated or that Wolbachia frequencies have increased since that study, neither of which is necessarily supported by the present data. When only female mosquitoes are considered using conventional PCR, the interpretation is considerably different. This distinction should be clearly explained to avoid overinterpretation.

Regarding my previous comment #7, I also believe the manuscript would benefit from a broader discussion on the management implications of areas where Wolbachia frequencies remain low or absent. Such "pockets" may represent barriers to the long-term stability of Wolbachia invasion and deserve consideration in terms of surveillance and possible supplementary release strategies.

Specific comments

l55. Correct the frequency with 60%

l367. Exclude "only".

l413. Replace "have been shown to" with "were"

l418-420. The sentence is confusing, since it seems that lab-reared adults had less Wolbachia than field-collected ones, while the opposite is true.

l473. Although I agree that pooled samples may provide overestimation, I can't see how they can understimate Wolbachia positivity.

Lines 477–481. I still believe it is important to emphasize that caution is needed when interpreting mosquitoes that are positive by qPCR but negative by conventional PCR. These individuals likely harbor lower Wolbachia densities, which may be associated with imperfect maternal transmission and reduced arbovirus blocking. This point has important implications for evaluating the long-term success of Wolbachia establishment.

Lines 495–498. An additional explanation worth discussing is that ovitraps negative for Wolbachia along the outskirts of the city may reflect the greater difficulty of maintaining Wolbachia-infected populations in areas that experience continuous immigration of uninfected mosquitoes from neighboring municipalities.

Lines 524–533. I respectfully disagree with the authors' interpretation that these findings should be viewed primarily with caution because of potential effects of mosquito age or nutritional status. Regardless of the mechanisms underlying the lower Wolbachia densities, the biological consequence remains the same: a higher proportion of females with undetectable or low-density Wolbachia implies less efficient maternal transmission and potentially weaker population-level arbovirus blocking. Therefore, I believe the implications of this result deserve stronger emphasis rather than greater caution.

l753. Coorect the uppercase letters in the author names and title.

l801. Correct "mosquitoes"

Reviewer #3: - The introduction is much improved in this version, and more clearly lays out the overall results and conclusions.

- Table 1: could you provide more commentary in the discussion on why the ovitrap prevalence estimates vary from the adult estimates? Does this change if you look at the same houses with both ovitraps and adults collected -- within a house are the estimates similar?

**********

PLOS authors have the option to publish the peer review history of their article (what does this mean?). If published, this will include your full peer review and any attached files.

If you choose “no”, your identity will remain anonymous but your review may still be made public.

**Do you want your identity to be public for this peer review?**  For information about this choice, including consent withdrawal, please see our Privacy Policy.

Reviewer #1: **Yes:**  Eric Caragata

Reviewer #2: No

Reviewer #3: No

**Figure resubmission:**

**Reproducibility:**

To enhance the reproducibility of your results, we recommend that authors of applicable studies deposit laboratory protocols in protocols.io, where a protocol can be assigned its own identifier (DOI) such that it can be cited independently in the future. Additionally, PLOS ONE offers an option to publish peer-reviewed clinical study protocols. Read more information on sharing protocols at https://plos.org/protocols?utm_medium=editorial-email&utm_source=authorletters&utm_campaign=protocols

# Author comment: Author response to Decision Letter 2

**DOI:** 10.1371/journal.pntd.0014630.r004

23 Jul 2026

Attachment Submitted filename: Response letter Revison2 Final.docx

# Editor report: Decision Letter 2

**DOI:** 10.1371/journal.pntd.0014630.r005

**Authors:** Audrey Lenhart, James Crainey

29 Jul 2026

Dear Dr. Avila,

We are pleased to inform you that your manuscript 'Wolbachia prevalence in Aedes aegypti across five comunas of Medellín, Colombia: implications for post-release surveillance' has been provisionally accepted for publication in PLOS Neglected Tropical Diseases.

Before your manuscript can be formally accepted you will need to complete some formatting changes, which you will receive in a follow up email. A member of our team will be in touch with a set of requests.

Please note that your manuscript will not be scheduled for publication until you have made the required changes, so a swift response is appreciated.

IMPORTANT: The editorial review process is now complete. PLOS will only permit corrections to spelling, formatting or significant scientific errors from this point onwards. Requests for major changes, or any which affect the scientific understanding of your work, will cause delays to the publication date of your manuscript.

Should you, your institution's press office or the journal office choose to press release your paper, you will automatically be opted out of early publication. We ask that you notify us now if you or your institution is planning to press release the article. All press must be co-ordinated with PLOS.

Thank you again for supporting Open Access publishing; we are looking forward to publishing your work in PLOS Neglected Tropical Diseases.

Best regards,

James Lee Crainey, Ph.D.

Academic Editor

PLOS Neglected Tropical Diseases

Audrey Lenhart

Section Editor

PLOS Neglected Tropical Diseases

Shaden Kamhawi

co-Editor-in-Chief

PLOS Neglected Tropical Diseases

orcid.org/0000-0003-4304-636XX

Paul Brindley

co-Editor-in-Chief

PLOS Neglected Tropical Diseases

orcid.org/0000-0003-1765-0002

***********************************************************

p.p1 {margin: 0.0px 0.0px 0.0px 0.0px; line-height: 16.0px; font: 14.0px Arial; color: #323333; -webkit-text-stroke: #323333}span.s1 {font-kerning: none

# Editor report: Acceptance letter

**DOI:** 10.1371/journal.pntd.0014630.r006

**Authors:** Audrey Lenhart, James Crainey

Dear Dr. Avila,

We are delighted to inform you that your manuscript, "Wolbachia prevalence in Aedes aegypti across five comunas of Medellín, Colombia: implications for post-release surveillance," has been formally accepted for publication in PLOS Neglected Tropical Diseases.

We have now passed your article onto the PLOS Production Department who will complete the rest of the publication process. All authors will receive a confirmation email upon publication.

The corresponding author will soon be receiving a typeset proof for review, to ensure errors have not been introduced during production. Please review the PDF proof of your manuscript carefully, as this is the last chance to correct any scientific or type-setting errors. Please note that major changes, or those which affect the scientific understanding of the work, will likely cause delays to the publication date of your manuscript. Note: Proofs for Front Matter articles (Editorial, Viewpoint, Symposium, Review, etc...) are generated on a different schedule and may not be made available as quickly.

Soon after your final files are uploaded, the early version of your manuscript will be published online unless you opted out of this process. The date of the early version will be your article's publication date. The final article will be published to the same URL, and all versions of the paper will be accessible to readers.

For Research Articles, you will receive an invoice from PLOS for your publication fee after your manuscript has reached the completed accept phase. If you receive an email requesting payment before acceptance or for any other service, this may be a phishing scheme. Learn how to identify phishing emails and protect your accounts at https://explore.plos.org/phishing.

Thank you again for supporting open-access publishing; we are looking forward to publishing your work in PLOS Neglected Tropical Diseases.

Best regards,

Shaden Kamhawi

co-Editor-in-Chief

PLOS Neglected Tropical Diseases

Paul Brindley

co-Editor-in-Chief

PLOS Neglected Tropical Diseases
